# Myeloid neddylation targets IRF7 and promotes host innate immunity against RNA viruses

**DOI:** 10.1371/journal.ppat.1009901

**Published:** 2021-09-10

**Authors:** Min Zhao, Yaolin Zhang, Xiqin Yang, Jiayang Jin, Zhuo Shen, Xiaoyao Feng, Tao Zou, Lijiao Deng, Daohai Cheng, Xueting Zhang, Cheng Qin, Chunxiao Niu, Zhenjie Ye, Xueying Zhang, Jia He, Chunmei Hou, Ge Li, Gencheng Han, Qianqian Cheng, Qingyang Wang, Lin Wei, Jie Dong, Jiyan Zhang

**Affiliations:** 1 Beijing Institute of Basic Medical Sciences, Beijing, China; 2 Department of Pathogen Biology, Hebei Medical University, Shijiazhuang, Hebei, China; 3 Chinese Institute for Brain Research, Beijing, China; University of Tennessee Health Science Center, UNITED STATES

## Abstract

Neddylation, an important type of post-translational modification, has been implicated in innate and adapted immunity. But the role of neddylation in innate immune response against RNA viruses remains elusive. Here we report that neddylation promotes RNA virus-induced type I IFN production, especially IFN-α. More importantly, myeloid deficiency of UBA3 or NEDD8 renders mice less resistant to RNA virus infection. Neddylation is essential for RNA virus-triggered activation of *Ifna* gene promoters. Further exploration has revealed that mammalian IRF7undergoes neddylation, which is enhanced after RNA virus infection. Even though neddylation blockade does not hinder RNA virus-triggered IRF7 expression, IRF7 mutant defective in neddylation exhibits reduced ability to activate *Ifna* gene promoters. Neddylation blockade impedes RNA virus-induced IRF7 nuclear translocation without hindering its phosphorylation and dimerization with IRF3. By contrast, IRF7 mutant defective in neddylation shows enhanced dimerization with IRF5, an *Ifna* repressor when interacting with IRF7. In conclusion, our data demonstrate that myeloid neddylation contributes to host anti-viral innate immunity through targeting IRF7 and promoting its transcriptional activity.

## Author summary

With the features of high mutation rates and fast propagation, RNA viruses remain a great challenge for the control and prevention of epidemic. Better understanding of the molecular mechanisms involved in host innate immunity against RNA viruses will facilitate the development of anti-viral drugs and vaccines. Neddylation has been implicated in innate and adapted immunity. But the role of neddylation in RNA virus-triggered type I IFN production remains elusive. Here, using mouse models with myeloid deficiency of UBA3 or NEDD8, we report for the first time that neddylation contributes to innate immunity against RNA viruses in mammals. Neddylation is indispensable for RNA virus-induced IFN-α production although its role in IFN-β production is much blunted in macrophages. In mechanism, neddylation directly targets IRF7 and enhances its transcriptional activity through, at least partially, promoting its nuclear translocation and preventing its dimerization with IRF5, an *Ifna* repressor when interacting with IRF7. Our study provides insight into the regulation of IRF7 and innate immune signaling.

## Introduction

Innate immunity is the body’s first line of defense against the invasion of viruses, of which macrophages are key components to inhibit the invasion and replication of viruses. After the recognition of viral nucleic acids, interferon regulatory factors (IRFs, mainly IRF3 and IRF7), nuclear factor κB (NF-κB), and activating protein 1 (AP1) are activated by innate immune signaling to induce the production of type I interferons (IFNs, IFN-α and IFN-β) and inflammatory cytokines [1–4]. Under steady state, unphosphorylated IRF3 and IRF7 stay in the cytoplasm and NF-κB is sequestered in the cytoplasm by inhibitor of κB (IκB) proteins. Upon phosphorylated by the IκB kinase (IKK)-related kinases, TANK-binding kinase 1 (TBK1) and IKKi, IRF3 and IRF7 undergo dimerization and nuclear translocation [5–8]. Phosphorylated IκB proteins undergo ubiquitin-mediated degradation, thereby releasing NF-κB. NF-κB then undergoes nuclear translocation and collaborates with IRFs to mediate gene transcription [4,9]. In most cell types including macrophages, IRF3 is constitutively expressed while IRF7 is expressed at a low level under steady state but is strongly induced by type I IFNs [8].

The roles of IRF3 and IRF7 in type I IFN production depend on the virus and the type of infected cells. In response to local influenza A infection, whole-body deletion of IRF7 completely abolished IFN-α production in the lung but only partially impaired early phase IFN-β production, whereas deletion of IRF3 significantly abrogated IFN-β production but only partially impaired early phase IFN-α production [10]. In mouse embryonic fibroblasts (MEFs), the potent induction of both IFN-α and IFN-β at various time points after herpes simplex virus (HSV)-1 or vesicular stomatitis virus infection was markedly blocked in the absence of IRF7, whereas IRF3 deficiency showed much attenuated effects, especially for IFN-α [11]. As for myeloid cells, IRF7 also plays a more pivotal than IRF3 in virus-triggered production of type I IFNs [11,12]. Intriguingly, virus-triggered production of IFN-β in macrophages seems to be much less IRF3 and IRF7-dependent than that of IFN-α. West Nile virus-triggered production of IFN-α in macrophages and myeloid dendritic cells (mDCs) was almost completely blocked in the absence of both IRF3 and IRF7 while that of IFN-β was only partially affected at early stage [13]. The separate regulation of IFN-α and IFN-β expression was also observed in human immunodeficiency virus (HIV)-infected macrophages [14].

The ubiquitination of phosphorylated IκB proteins is carried out by Skp1/Cullin1/F-box protein β-TrCP (SCF^β-TrCP^) ubiquitin-E3 ligase complex [15–17]. The activity of SCF-like ubiquitin-E3 ligase complexes is promoted by neddylation. Neddylation is an important type of post-translational modification, involving an enzymatic reaction by which a ubiquitin-like protein, neural precursor cell-expressed, developmentally down-regulated 8 (NEDD8), is covalently conjugated to the substrate [18]. The only known NEDD8-activating enzyme E1 (NAE) is a heterodimer comprising scaffold amyloid precursor protein binding protein 1 (APPBP1) and catalytic subunit ubiquitin-like modifier activating enzyme 3 (UBA3). Then the NEDD8-loaded NAE transfers NEDD8 to the NEDD8-conjugating enzyme E2 (usually Ubc12) through a trans-thiolation reaction. Ultimately, NEDD8 is targeted to its substrate protein via covalent attachment by certain E3 ligase(s) [19–23]. The best-characterized substrates of neddylation are Cullins. The neddylation of Cullins stimulates the ubiquitination activity of the E3 complex and thereby contributes to NF-κB activation. Intriguingly, neddylation blockade by treatment with NAE inhibitor MLN4924 or depletion of *Uba3* delayed but did not completely block DNA virus HSV-1-induced nuclear translocation of NF-κB. One the other hand, neddylation inhibition showed no effects on HSV-1-induced IRF3 phosphorylation and nuclear translocation. Consequently, MLN4924 treatment or UBA3 deficiency negatively regulated HSV-1-induced early phase IFN-β production from bone marrow-derived macrophages (BMDMs) [24].

As for RNA viruses, NF-κB contributes to IFN-β production at early phase of RNA virus infection when IRF3 activation is weak [25]. On the other hand, it has been reported that Sendi virus (SeV) infection induced the degradation of IRF3 through Cullin 1-based ubiquitination [26]. Theoretically, neddylation should promote SeV-induced NF-κB activation but simultaneously inhibit IRF3 activation. The paradox might result in different outcomes in different types of cells. One group has reported that NEDD8 knockdown in HeLa, HEK-293T, and THP-1 cells showed no effect on IFN-β production triggered by lipopolysaccharide (LPS), poly (I:C), or SeV even though MLN4924 potently inhibited it [27]. In zebrafish, MLN4924 treatment or disruption of *Nedd8* increased the sensitivity of zebrafish to RNA virus Spring Viremia of Carp Virus infection, which was associated with diminished expression of type I IFNs and interferon-stimulated genes (ISGs) [28]. The neddylation of zebrafish IRF3 and IRF7 was detected under the conditions of co-overexpression with NEDD8 in HEK-293T cells [28]. Thus, the role of neddylation in RNA virus-triggered type I IFN production should be double checked. Whether IRF3 and IRF7 are genuine neddylation targets remains to be clarified.

Here, using mouse models with myeloid deficiency of UBA3 or NEDD8, we report that myeloid neddylation is indispensable for RNA virus-induced production of type I IFNs, especially IFN-α. In mechanism, neddylation directly targets IRF7 and enhances its transcriptional activity through, at least partially, promoting its nuclear translocation and preventing its dimerization with IRF5, an *Ifna* repressor when interacting with IRF7.

## Results

### Neddylation promotes RNA virus-induced IFN-α production in myeloid cells

To investigate the potential role of neddylation in RNA virus-induced type I IFN production, mice with myeloid deficiency of NEDD8 (*Nedd8*^F/F; Lyz2-Cre^, named as *Nedd8*^ΔMye^) as well as UBA3 (*Uba3*^F/F; Lyz2-Cre^, named as *Uba3*^ΔMye^) [24] were generated. Even though neddylation suppression by MLN4924 treatment was reported to result in the apoptosis of macrophages *in vitro* [29], flow cytometry analysis revealed that *Uba3*^ΔMye^ and *Nedd8*^ΔMye^ mice and their control (*Uba3*^F/F^ and *Nedd8*^F/F^) littermates exhibited similar percentages of F4/80^+^CD11b^+^ macrophages in the peripheral blood, bone marrow, spleen, and peritoneal cavity (S1 Fig). Thus, neddylation is dispensable for the development and survival of macrophages under steady state.

**Fig 1:**
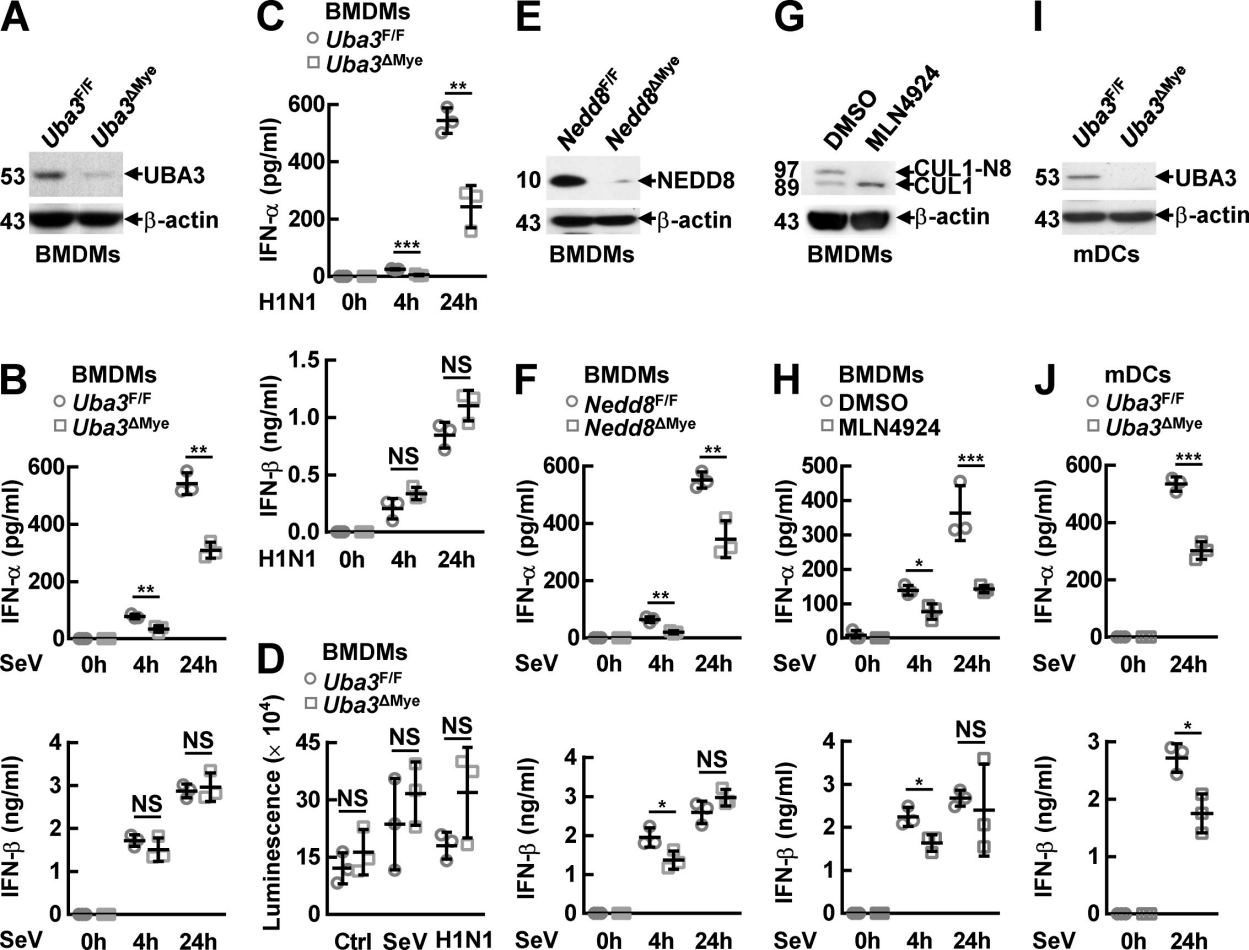
Neddylation promotes RNA virus-induced IFN-α production in myeloid cells. (A-F, I, J) BMDMs or mDCs were cultured from the indicated mouse models. After immunoblotting (IB) analysis with the indicated antibodies to confirm the deficiency of UBA3 (A, I) or NEDD8 (E), these myeloid cells were infected with the indicated RNA viruses for the indicated time periods. Then the supernatants were subjected to ELISA (B, C, F, J). The survival of UBA3-deficient BMDMs before and 24 h after RNA virus infection was measured by ATP lite assays (D). (G) IB analysis to confirm the efficiency of MLN4924 pretreatment (0.1 μM, 3 h) in wild type (WT) BMDMs. (H) After MLN4924 pretreatment (0.1 μM, 3 h), WT BMDMs were infected with SeV for the indicated time periods. Then the supernatants were subjected to ELISA. Quantitative data are shown as Mean ± SD (*n* = 3 per group). *p< 0.05; **p< 0.01; ***p< 0.001; NS, not significant. CUL-N8, neddylated Cullin 1.

We then cultured BMDMs from*Uba3*^ΔMye^ mice and their control littermates. Immunoblotting (IB) analysis confirmed UBA3 deficiency (Fig 1A). After infection with SeV or influenza A H1N1 virus for different time periods, ELISA assays of the supernatants revealed that both early phase (4 h) and late phase (24 h) IFN-α production was significantly impaired in BMDMs from *Uba3*^ΔMye^ mice, as compared to those from littermate control mice (Fig 1B and 1C). The early phase effect seems more pronounced than the late phase. However, there was no statistically significant difference in IFN-β production, either at early phase (4 h) or at late phase (24 h) of RNA virus infection (Fig 1B and 1C). The survival of UBA3-deficient BMDMs was not significantly affected before and 24 h after RNA virus infection (Fig 1D). For further confirmation, we cultured BMDMs from *Nedd8*^ΔMye^ mice and their control littermates. BMDMs from wild type (WT) mice were also cultured and treated with MLN4924. IB analysis confirmed NEDD8 deficiency (Fig 1E) or the blockade of Cullin 1 (CUL1) neddylation (Fig 1G). The two strategies of neddylation blockade in BMDMs also led to significantly impaired IFN-α production at both early phase (4 h) and late phase (24 h) of SeV infection, which was associated with partially reduced IFN-β production at early phase (4 h) but not at late phase (24 h) (Fig 1F and 1H). Thus, neddylation is indispensable for RNA virus-induced IFN-α production in macrophages but its role in IFN-β production is relatively weak, especially in the case of UBA3 deficiency. In this scenario, we also cultured mDCs from *Uba3*^ΔMye^ mice and their control littermates (Fig 1I). ELISA assays revealed that UBA3 deficiency resulted in diminished production of IFN-β as well as diminished IFN-α even at late phase (24 h) of SeV infection (Fig 1J), suggesting that RNA virus-triggered IFN-β production in mDCs is more sensitive to neddylation blockade than that in BMDMs. In addition, MLN4924 pretreatment significantly abrogated the production of IFN-α and IFN-β in MEFs both at early phase (4 h) and at late phase (24 h) of SeV infection (S2 Fig).

### Myeloid neddylation blockade renders mice less resistant to RNA virus infection

**Fig 2:**
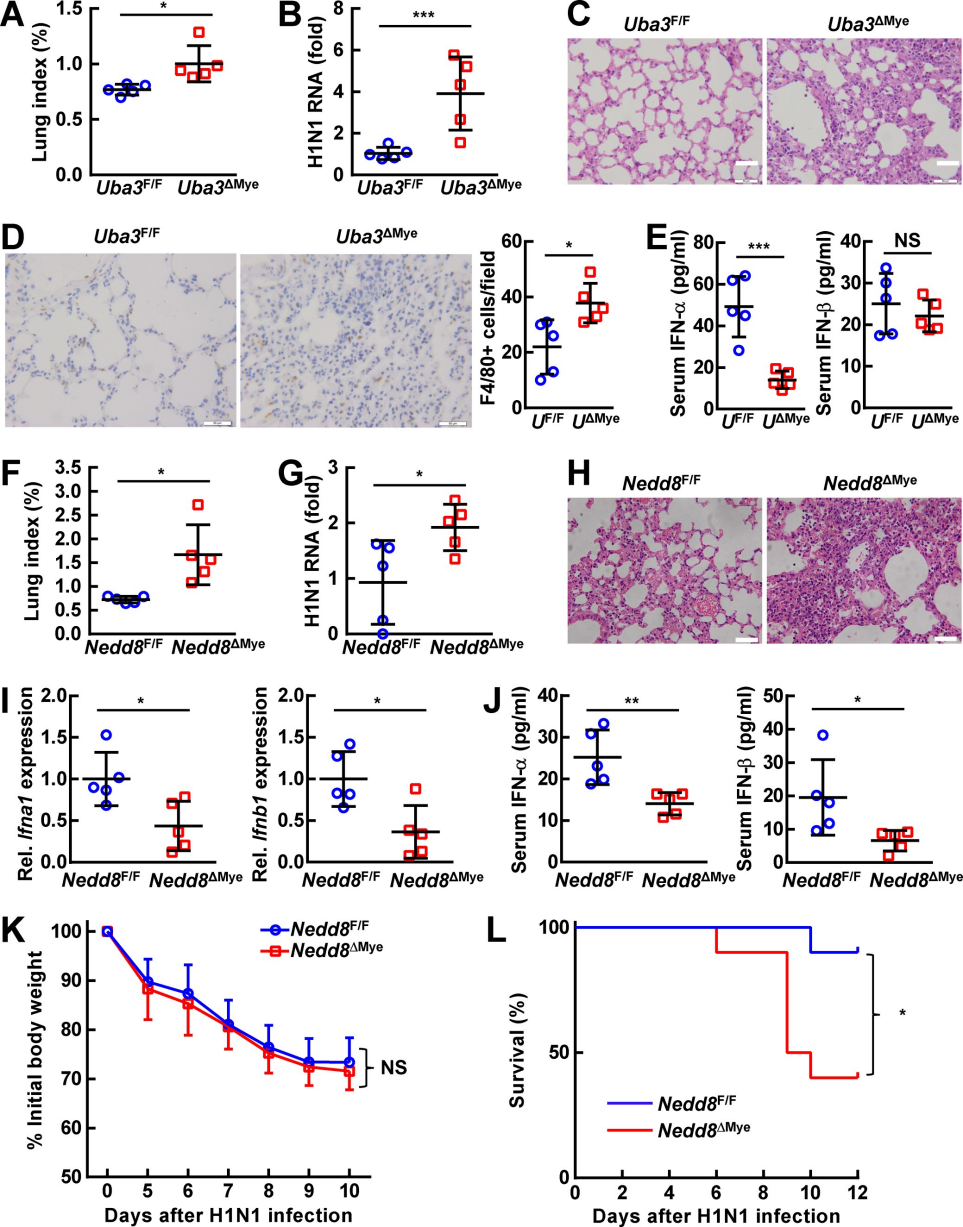
Myeloid neddylation blockade renders mice less resistant to RNA virus infection. 8-week-old*Uba3*^F/F^ and *Uba3*^ΔMye^ mice (A-E) or *Nedd8*^F/F^ and *Nedd8*^ΔMye^ mice (F-L) were challenged intranasally with 500 PFU Influenza A/Puerto Rico/8/1934 H1N1 virus. Mice were sacrificed and the serum and lung tissues were collected 24 h (A-E, *n* = 5 per group) or 7 days (F-J, *n* = 5 per group) later. (A, F) The lung index was determined by calculating lung weight relative to body weight. (B, G) Levels of H1N1 RNA in lung tissues were analyzed by quantitative RT-PCR analysis. (C, H) Histopathology of lung tissues was analyzed by H & E staining. (D) The number of macrophages in lung tissues was determined by immunohistochemistry staining of surface marker F4/80. (E, J) Levels of IFN-α and IFN-β in the serum were measured by ELISA. (I) Levels of *Ifna1* and *Ifnb1* mRNA in lung tissues were analyzed by quantitative RT-PCR analysis. (K, L) In another round of infection, body weight changes and survival curves were monitored at different time points after H1N1 infection (*n* = 10 per group). Quantitative data are shown as Mean ± SD. *p< 0.05; **p< 0.01; ***p< 0.001; NS, not significant.

Alveolar macrophages are reported to be the first defense line in response to local RNA virus infection [30]. Since myeloid neddylation promotes RNA virus-induced production of type I IFNs, especially IFN-α, we infected *Uba3*^ΔMye^and *Nedd8*^ΔMye^mice and their control littermates intranasally with influenza A H1N1 virus [31]. The lung tissues and serum of *Uba3*^ΔMye^ mice were firstly harvested at 24 h after infection. Compared with control littermates, the lung index and the viral burden were significantly higher in *Uba3*^ΔMye^ mice (Fig 2A and 2B). Accordingly, *Uba3*^ΔMye^ lungs showed exaggerated epithelial damage and parenchymal infiltration of inflammatory cells (Fig 2C). Indeed, immunohistochemistry analysis revealed more F4/80-positive macrophages in *Uba3*^ΔMye^ lungs after H1N1 challenge (Fig 2D). ELISA revealed lower levels of IFN-α and comparable levels of IFN-β in the serum of *Uba3*^ΔMye^ mice, as compared to littermate *Uba3*^F/F^ mice (Fig 2E).

To evaluate the effects of myeloid neddylation blockade on the disease and survival of infected mice over time, we harvested the lung tissues and serum of *Nedd8*^ΔMye^ mice 7 days after infection. As expected, the lung index and the viral burden were significantly higher in *Nedd8*^ΔMye^ mice at this time point (Fig 2F and 2G), which was associated with exaggerated pulmonary inflammation (Fig 2H). Quantitative RT-PCR revealed lower levels of *Ifna1* and *Ifnb1* mRNA in *Nedd8*^ΔMye^ lung tissues (Fig 2I). Furthermore, ELISA confirmed lower levels of both IFN-α and IFN-β in the serum of *Nedd8*^ΔMye^ mice (Fig 2J). In this scenario, we assessed whether neddylation blockade can affect the mortality in another round of infection. Even though myeloid NEDD8 deficiency failed to aggravate the reduction of the body weight (Fig 2K), more *Nedd8*^ΔMye^ mice died during the 12-day observation period (Fig 2L). Thus, myeloid neddylation blockade renders mice less resistant to RNA virus infection.

### Neddylation promotes RNA virus-triggered activation of *Ifna* promoters

**Fig 3:**
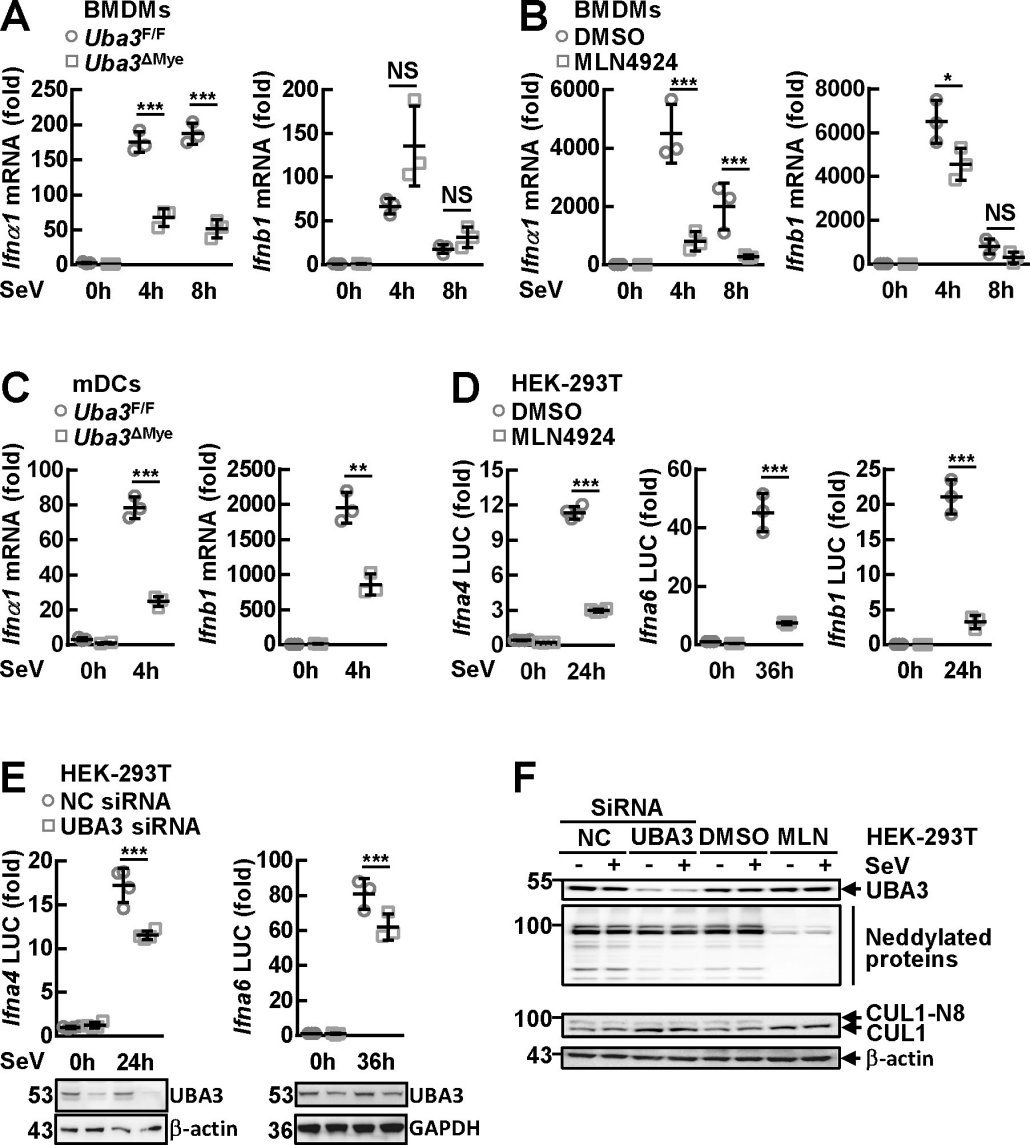
Neddylation promotes the activation of *Ifna* promoters by RNA virus. (A-C) After BMDMs (A) or mDCs (C) were cultured from *Uba3*^F/F^ and *Uba3*^ΔMye^ mice or WT BMDMs were pretreated MLN4924 (0.1 μM, 3 h) (B), the cells were infected with SeV for the indicated time periods. Then cells were subjected to quantitative RT-PCR analysis. (D-F) Twenty-four hours after HEK-293T cells were transfected with the indicated reporter plasmids in the presence or absence of the indicated small interfering RNAs (siRNAs), the cells were pretreated with or without MLN4924 (0.5 μM, 3 h), followed by SeV infection for the indicated time periods. Dual-reporter luciferase (LUC) assays were then performed. Luciferase activity was reported as fold induction (D and *Top* part of E). Cell lysates were subjected to IB with the indicated antibodies (*Bottom* part of E and F). Quantitative data are shown as Mean ± SD (*n* = 3 ~ 4 per group). *p< 0.05; **p< 0.01; ***p< 0.001; NS, not significant.

In this scenario, we tried to analyze how neddylation might affect type I IFN production at the transcription level. Quantitative RT-PCR revealed that the upregulation of *Ifna1* in BMDMs after SeV infection for 4 h or 8 h diminished upon UBA3 deficiency (Fig 3A) or MLN4924 pretreatment (Fig 3B). However, the upregulation of *Ifnb1* was not suppressed upon UBA3 deficiency (Fig 3A) although it was slightly reduced at 4 h post-infection upon MLN4924 pretreatment (Fig 3B). In mDCs, SeV-induced upregulation of both *Ifna1* and *Ifnb1* diminished in the absence of UBA3 (Fig 3C). Thus, the changes in type I IFN mRNA levels are consistent with those in IFN-α and IFN-β secretion levels (Fig 1). Next, we tried to analyze the effects of neddylation inhibition on RNA virus-triggered promoter activation in HEK-293T cells. Dual-reporter luciferase assays revealed that *Ifna4* and *Ifnb1* promoters were significantly activated after SeV infection for 24 h whereas the activation of *Ifna6* promoter, which solely depends on IRF7 [32], only occurred after prolonged SeV infection. The induction of all the three promoters by SeV infection was abrogated by MLN4924 pretreatment (Fig 3D). Because in macrophages neddylation is more important for RNA virus-triggered induction of IFN-α than for that of IFN-β, we decided to focus on *Ifna* gene promoters. We further tested the effects of neddylation inhibition on *Ifna* promoter activation with the strategy of RNA interference. As expected, UBA3 knockdown partially inhibited the induction of both *Ifna4* promoter and *Ifna6* promoter by SeV infection (Fig 3E). The weaker effects of UBA3 small-interfering RNA (siRNA), as compared to MLN4924 pretreatment, were consistent with the lower efficiency of neddylation inhibition. MLN4924 was very efficient to block both global neddylation and Cullin 1 neddylation, while the global neddylation and the ratio of neddylated Cullin 1 to free Cullin 1 were only partially reduced upon UBA3 knockdown (Fig 3F).

### Mammalian IRF7 is a neddylation substrate

**Fig 4:**
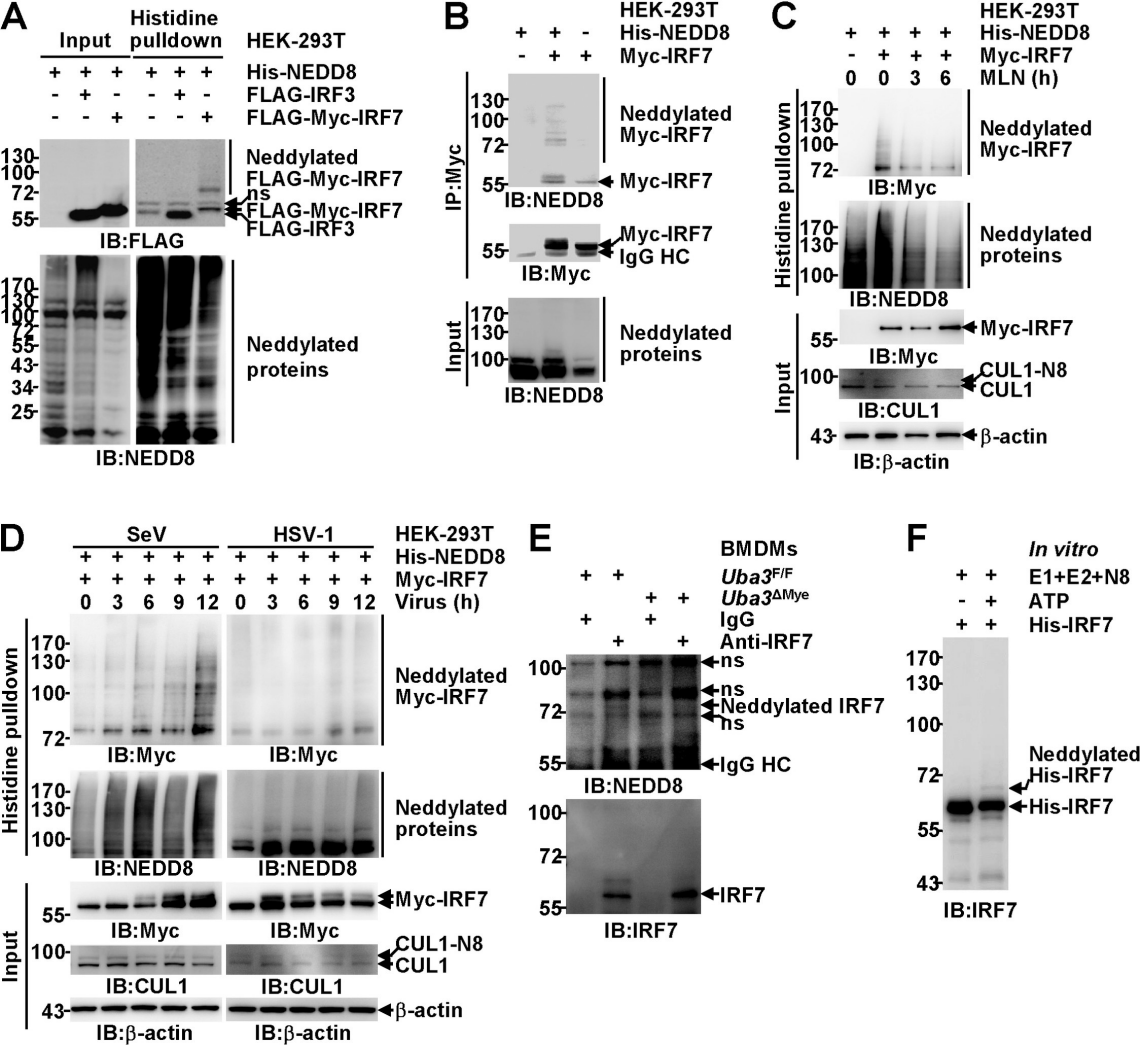
Mammalia IRF7 is a neddylation substrate. (A, B) HEK-293T cells were transfected with the indicated mammalian expression vectors. Twenty-four hours later, possible neddylation of exogenous mammalian IRF proteins was examined by IB analysis with the indicated antibodies after histidine pulldown under fully denaturing conditions (A) or immunoprecipitation (IP) under partially denaturing conditions with an antibody against Myc-tag (B). (C, D) HEK-293T cells were transfected with the indicated mammalian expression vectors. Twenty-four hours later, the cells were treated with 0.5 μM MLN4924 (C) or the indicated viruses (D) for the indicated time periods. The neddylation of exogenous murine IRF7 was then examined by histidine pulldown under fully denaturing conditions. (E) After BMDMs from *Uba3*^F/F^ and *Uba3*^DMye^ mice were infected with SeV for 6 h, the neddylation of endogenous IRF7 was then examined by IB with the indicated antibodies after IP with an anti-IRF7 antibody. (F) Bacterially expressed His-tagged murine IRF7 was incubated with the indicated purified proteins at 37°C in the absence or presence of ATP for 1 h. The samples were then subjected to IB with an antibody against IRF7.

Our aforementioned data suggest that neddylation promotes RNA virus-triggered IFN-α expression or *Ifna* promoter activation in all the cell types tested (Figs 1,3 and S2). However, its role in IFN-β induction is much blunted in macrophages (Figs 1, 3 and S2). These findings echo a previous report that IRF3 and IRF7 are indispensable for virus-induced IFN-α production but only partially contribute to early stage IFN-β expression in macrophages [13]. Because zebrafish IRF3 and IRF7 have been reported as potential neddylation substrates [28], we further explored whether mammalian IRF3 and IRF7 are neddylation substrates. With the strategy of co-transfection in HEK-293T cells, histidine pulldown under fully denaturing conditions clearly demonstrated that His-tagged NEDD8 was covalently conjugated to exogenous murine IRF7. However, no modification of exogenous human IRF3 was detected under the same conditions (Fig 4A). Unmodified versions of exogenous IRF3 and IRF7 were also detected in the pulldown products (Fig 4A), possibly due to non-specific electrostatic attraction to the nickel beads. Moreover, by performing immunoprecipitation (IP) under partially denaturing conditions, we found exogenous IRF7 in the precipitates was detected as smear bands by an antibody against NEDD8 (Figs 4B and S3). Unmodified versions of exogenous IRF7 were also detected by the anti-NEDD8 antibody, possibly due to non-specific recognition of too many immunoprecipitated proteins (Figs 4B and S3). Both strategies of *in vivo* neddylation assays indicate the molecular weight of the major modification band was about 16-20kDa higher than free tagged IRF7 (Figs 4A,4B and S3).

NEDD8 overexpression might lead to artificial conjugation independent of NAE [33]. Because histidine pulldown under fully denaturing conditions revealed that covalent modification of exogenous murine IRF7 was reduced after MLN4924 treatment for 3 h and was further eliminated after MLN4924 treatment for 6 h, the neddylation of exogenous IRF7 was not artificial (Fig 4C). Next, we examined whether viral infection affects IRF7 neddylation. SeV significantly enhanced the neddylation of exogenous murine IRF7 in a time-dependent manner, whereas HSV-1 showed no effect at 3 h and 6 h post-infection although it slightly enhanced IRF7 neddylation at 9 h and 12 h post-infection. Both viruses potently induced an upshifted band of IRF7, indicating active IRF7 post translational modifications (Fig 4D). Notably, HSV-1-induced IRF7 expression in macrophages was very weak, whereas potent IRF7 induction occurred after SeV infection at the dose that HSV-1 and SeV induced similar NF-κB p65 phosphorylation at Ser536 (S4 Fig). SeV-induced IRF7 expression in macrophages was accompanied with two weak upshifted bands: one at around 60 kDa and another at the position similar to neddylated IRF7 (as indicated by the symbol <), suggesting that the neddylation of mammalian IRF7 could occur at the endogenous level. To confirm this notion, SeV-infected BMDMs from *Uba3*^F/F^ and *Uba3*^ΔMye^ mice were subjected to IP under partially denaturing conditions with an antibody against IRF7. Endogenous IRF7 in the precipitates of UBA3-sufficient BMDMs was detected as a major band at 56 kDa and an upshifted band at about 60 kDa by an anti-IRF7 antibody. However, no band around 72 kDa was detected, possibility due to the poor recognition of neddylated IRF7 by the anti-IRF7 antibody used for IP. Intriguingly, the upshifted band at about 60 kDa diminished in the absence of UBA3.When we used an antibody against NEDD8 for IB, endogenous IRF7 in the precipitates of UBA3-sufficient BMDMs was detected as a weak band slightly higher than 72 kDa after long exposure, which diminished upon UBA3 deficiency (Fig 4E).

In line with a recent study which indicated that the interaction between neddylation substrate and Ubc12 may be very difficult to be detected [34], the physiological interaction of IRF7 with Ubc12 was only weakly detected by IP endogenous proteins (S5 Fig). To prove a protein is a substrate for any type of enzyme-mediated post-translational modification, *in vitro* cell-free assays using this protein expressed and purified from bacteria or other non-eukaryotic cells/systems are the key approach. E3-independent NEDD8 covalent conjugation to several neddylation substrates (for example, p53, E2F1, and MKK7) *in vitro* has been demonstrated [20,35,36]. In this scenario, we analyzed whether the same phenomenon might happen to IRF7. Incubation of bacterially expressed His-tagged murine IRF7 with NAE (E1), Ubc12 (E2), and NEDD8 in the presence of ATP resulted in the appearance of a weak but repeatedly detectable slower-migrating band about 8–10 kDa higher than free tagged IRF7 (Fig 4F). Together, these data indicate that mammalian IRF7 is a neddylation substrate.

### Neddylation does not promote RNA virus-induced IRF7 expression

Then we set out to explore how neddylation might affect RNA virus-triggered innate immune signaling including IRF7 induction. IB analysis revealed an accumulation of Ser32 phosphorylated IκBα in UBA3-deficient, NEDD8-deficient, or MLN4924-pretreated BMDMs at 6 h and 8 h after SeV infection (S6A–S6C Fig). Since the phosphorylation of upstream kinase TBK1 at Ser172 and another TBK1 substrate NF-κB p65 at Ser536 was not augmented under the same conditions (S6A–S6C Fig), it is unlikely that enhanced IκBα phosphorylation at Ser32 upon neddylation blockade resulted from augmented upstream signaling. Rather, these data are consistent with the known role of neddylation in promoting SCF^β-TrCP^-mediated degradation of phosphorylated IκBα [15–17]. Indeed, SeV-induced IκBα degradation and p65 nuclear translocation were abrogated (S6A–S6C Fig and S7 Figs). In line with a previous report that a Cullin 1-based ubiquitin ligase is involved in SeV-induced IRF3 degradation [26], SeV-triggered IRF3 degradation was impaired in NEDD8-deficient or MLN4924-pretreated BMDMs, which was associated with enhanced IRF3 phosphorylation at Ser396 (S6B and S6C Fig). However, the reversal of IRF3 protein level in the absence of UBA3 was marginal although the increase in IRF3 phosphorylation at Ser396 reached statistical significance at 6 h after SeV infection (S6A Fig). Despite of the significantly defective IFN-α production, SeV-induced IRF7 expression was not hampered in UBA3-deficient, NEDD8-deficient, or MLN4924-pretreated BMDMs (S6A–S6C Fig).

### The transcriptional activity of mammalian IRF7 depends on covalent attachment of NEDD8 to its C-terminal lysines

**Fig 5:**
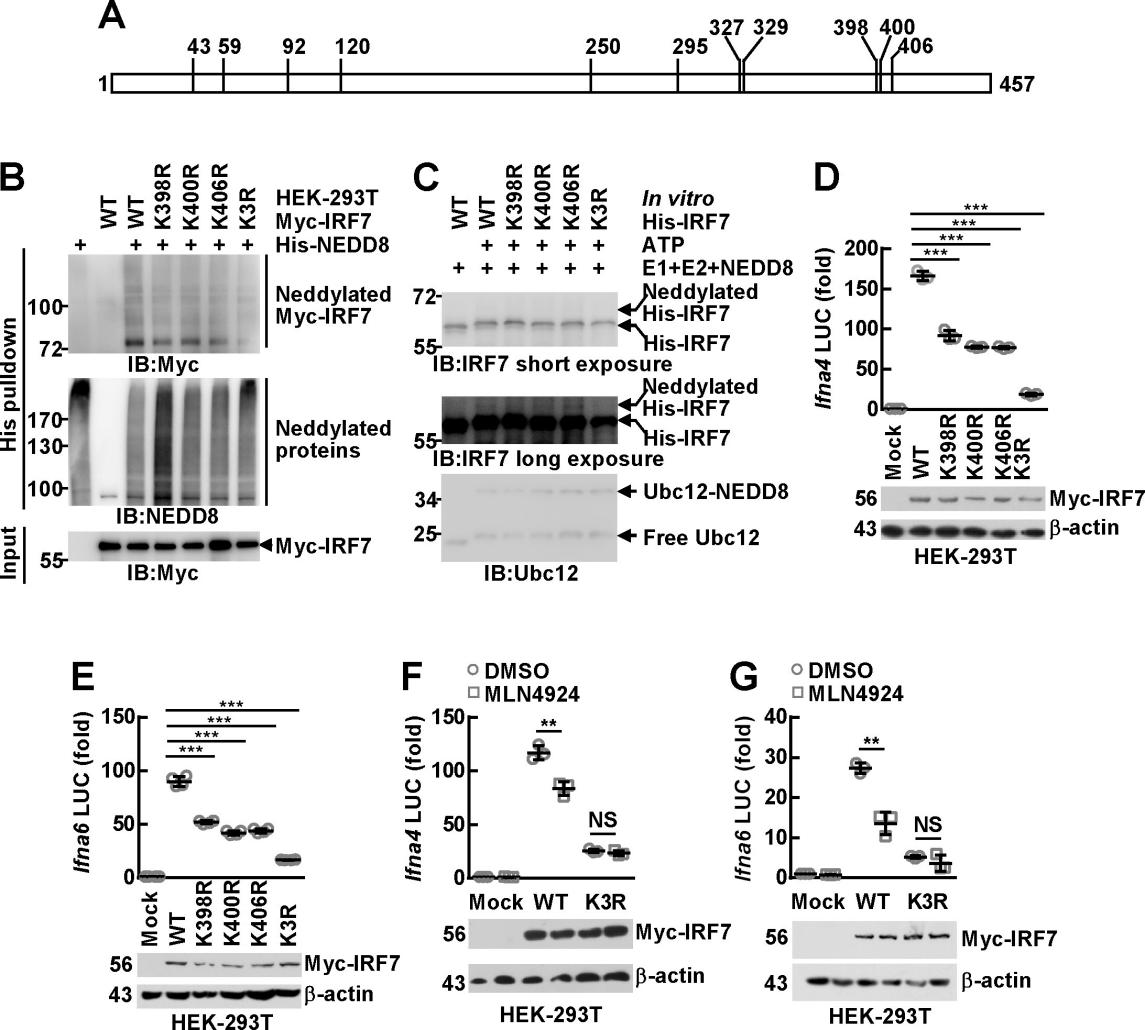
The transcriptional activity of mammalian IRF7 depends on covalent attachment of NEDD8 to its C-terminal lysines. (A) A schematic representation of all the lysine residues in murine IRF7. (B) HEK-293T cells were transfected with mammalian expression vectors encoding His-NEDD8 and FLAG-Myc-tagged murine IRF7 WT or mutants. Twenty-four hours later, the neddylation of exogenous murine IRF7 was examined by IB analysis with the indicated antibodies after histidine pulldown under fully denaturing conditions. (C) Bacterially expressed His-tagged murine IRF7 WT or mutants were subjected to *in vitro* neddylation (37°C, 1 h), followed by IB with the indicated antibodies. (D-G) FLAG-Myc-tagged murine IRF7 WT and mutants were co-transfected with reporter plasmids driven by *Ifna4* promoter or *Ifna6* promoter in HEK-293T cells. After 12 h, the cells were treated with 0.5μM MLN4924 for another 24 h or left untreated. Dual-reporter luciferase (LUC) assays were then performed with the supernatants. Luciferase activity was reported as fold induction (*Top*). Cell lysates were subjected to IB analysis with antibodies against Myc tag and β-actin (*Bottom*). Quantitative data are shown as Mean ± SD (*n* = 3 ~ 4 per group). **p< 0.01; ***p< 0.001; NS, not significant.

In order to study the function of neddylation on IRF7, it is necessary to identify the potential modification site(s). As neddylation occurs on specific lysine site(s), all lysines in murine IRF7 (Fig 5A) were individually mutated to arginines. His-tagged NEDD8 was co-transfected with FLAG-Myc-tagged WT murine IRF7 or mutants in HEK-293T cells. Histidine pulldown under fully denaturing conditions revealed that most mutation did not hinder murine IRF7 neddylation (S8 Fig), although K398R, K400R, and K406R mutants of murine IRF7 showed declined modification (Fig 5B). Indeed, mass spectrometry analysis of the precipitates obtained in S3 Fig revealed that peptides containing Lys398, Lys400, and Lys406 or Lys327 and Lys329 undergo either neddylation or ubiquitination (S9 Fig). Because mutation of Lys327 or Lys329 showed no inhibitory effect on murine IRF7 neddylation (S8 Fig), we decided to focus on Lys398, Lys400, and Lys406. Lysines that are near each other might play redundant roles as neddylation sites for certain NEDD8 substrates [20,37]. Therefore, we simultaneously mutated Lys398, Lys400, and Lys406 to arginines (K3R) for further examination. Indeed, the neddylation of K3R mutant was further suppressed (Fig 5B). The residual neddylation signals in the K3R mutant may come from Lys327 and Lys329, as suggested by mass spectrometry analysis (S9 Fig). We also employed the cell-free experimental system to pinpoint the neddylation sites. Although K398R, K400R, and K406R mutants exhibited similar modification, the neddylation of K3R mutant was significantly suppressed (Fig 5C). Therefore, the C-terminal lysines are the major neddylation sites for IRF7 despite that E2-mediated neddylation can easily occur as long as there are any neddylation site(s) in the substrate left.

In this scenario, FLAG-Myc-tagged murine IRF7 WT and mutants were co-transfected with reporter plasmids driven by *Ifna4* promoter or *Ifna6* promoter in HEK-293T cells. Dual-reporter luciferase assays revealed that both *Ifna4* promoter and *Ifna6* promoter were significantly activated by exogenous murine IRF7 (Fig 5D and 5E). However, K398R, K400R, and K406R mutants of murine IRF7 showed declined effects and K3R mutation almost completely abrogated the role of murine IRF7 (Fig 5D and 5E). Moreover, MLN4924 treatment significantly suppressed the activation of these promoters by murine IRF7 but its inhibitory effects diminished upon K3R mutation (Fig 5F and 5G). Together, these data suggest that the transactivation ability of IRF7 depends on covalent attachment of NEDD8 to its C-terminal lysines.

### The neddylation of mammalian IRF7 facilitates its nuclear translocation

**Fig 6:**
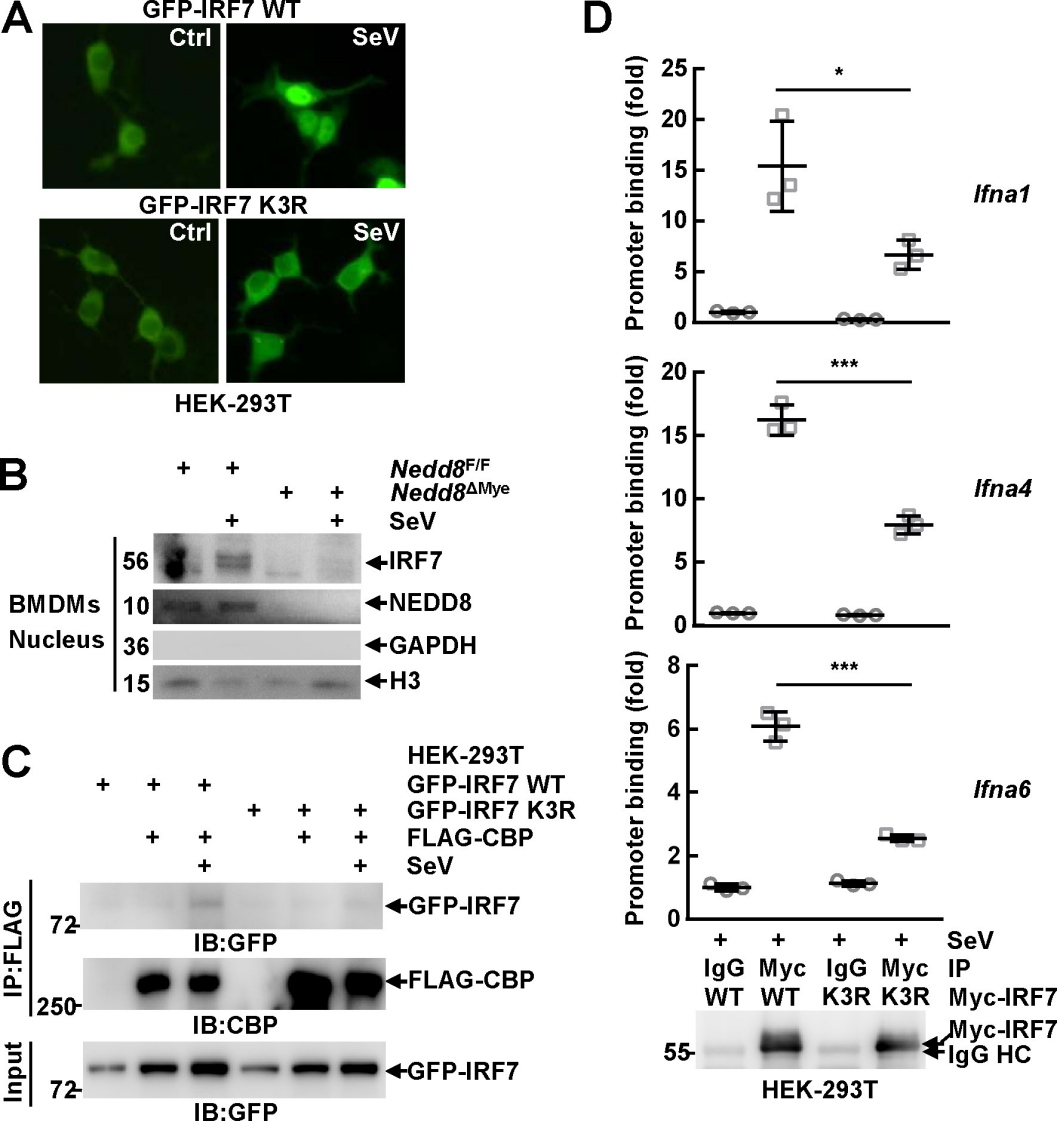
The neddylation of mammalian IRF7 facilitates its nuclear translocation. (A)Twenty-four hours after HEK-293T cells were transfected with mammalian expression vectors encoding GFP-tagged murine IRF7 WT and K3R mutant, the cells were infected with SeV for 6 h or left untreated. Then the subcellular localization of exogenous murine IRF7 was examined via confocal microscopy. (B) After BMDMs were cultured from *Nedd8*^F/F^ and *Nedd8*^ΔMye^ mice, the cells were infected with SeV for 6 h or left untreated. The subcellular localization of IRF7 was examined by nuclear cytoplasmic fractionation and subsequent IB. GAPDH was regarded as a cytoplasm marker and H3 as a nucleus marker. (C, D) Twenty-four hours after HEK-293T cells were transfected with the indicated mammalian expression vectors, the cells were infected with SeV for 6 h or left untreated. Then the interaction between exogenous murine IRF7 and exogenous murine CBP was determined by Co-IP (C). The specific binding of exogenous murine IRF7 to selected *Ifna* gene promoters within the chromatin was analyzed by ChIP with an anti-Myc antibody (D, *Top*). Immunoprecipitated exogenous murine IRF7 was analyzed by IB analysis (D, *Bottom*). Quantitative data are shown as Mean ± SD (*n* = 3 per group). *p< 0.05; ***p< 0.001.

The exact mechanism(s) by which the neddylation of IRF7 enhances its transcriptional activity are of interest. IRF7 must enter the nucleus to activate the transcription of target genes [1–3]. Unexpectedly, SeV-induced nuclear translocation of GFP-tagged murine IRF7 in HEK-293T cells was impaired upon K3R mutation (Fig 6A). In this scenario, NEDD8-sufficient and -deficient BMDMs before and after SeV infection for 6 h were subjected to nuclear cytoplasmic fractionation. IB analysis revealed the nuclear translocation of endogenous IRF7 at 6 h after SeV infection was impaired in the absence of NEDD8 (Fig 6B). In line with these observations, co-immunoprecipitation (Co-IP) analysis revealed that K3R mutation hindered SeV-induced interaction of murine IRF7 with nuclear transcriptional co-activator CBP [4,38] in HEK-293T cells (Fig 6C). Furthermore, chromatin immunoprecipitation (ChIP) assays demonstrated that murine IRF7 bound to different *Ifna* gene promoters in HEK-293T cells after SeV infection for 6 h and such ability diminished upon K3R mutation (Fig 6D). Thus, the neddylation of IRF7 enhances its transcriptional activity through, at least partially, promoting its nuclear translocation.

### The neddylation of mammalian IRF7 prevents its dimerization with IRF5

**Fig 7:**
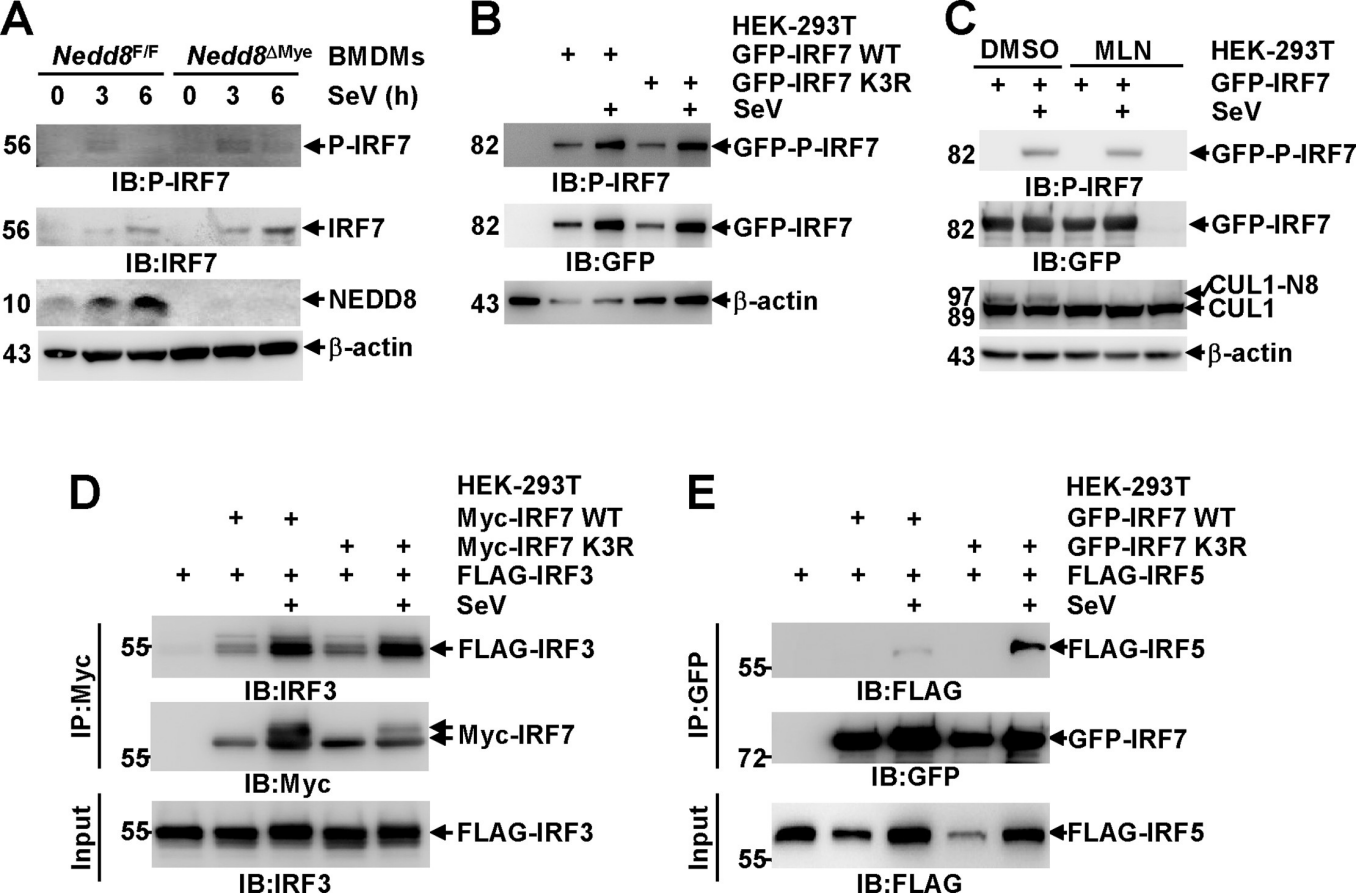
The neddylation of mammalian IRF7 prevents its dimerization with IRF5. (A-C) After BMDMs were cultured from *Nedd8*^F/F^ and *Nedd8*^ΔMye^ mice or 24 h after HEK-293T cells were transfected with the indicated mammalian expression vectors, the cells were pretreated with or without MLN4924 (0.5 μM, 3 h), followed by SeV infection for various periods of time as indicated. Then the phosphorylation and expression of murine IRF7 was detected by IB with the indicated antibodies. P-IRF7, phosphorylated IRF7 at Ser437/438. (D, E) Twenty-four hours after HEK-293T cells were transfected with the indicated mammalian expression vectors, the cells were infected with SeV for 6 h or left untreated. Then the interaction between exogenous murine IRF7 and exogenous human IRF3 (D) or exogenous human IRF5 (E) was determined by Co-IP.

A prerequisite for the nuclear translocation of IRF7 is its phosphorylation and dimerization [1–3]. To test how neddylation might affect IRF7 phosphorylation, BMDMs from *Nedd8*^F/F^ and *Nedd8*^ΔMye^ mice were infected with SeV for various periods of time. IB analysis revealed that NEDD8 deficiency did not hinder SeV-induced phosphorylation of endogenous murine IRF7 at Ser437/438 (Fig 7A). Notably, the antibody used to detect the phosphorylation of endogenous IRF7 only yielded weak signal. In this scenario, we also checked how neddylation blockade could affect the phosphorylation of exogenous IRF7 in HEK-293T cells. As expected, SeV-induced phosphorylation of exogenous murine IRF7 at Ser437/438 was not affected upon K3R mutation (Fig 7B) or MLN4924 pretreatment (Fig 7C). On the other hand, Co-IP analysis revealed that K3R mutation of IRF7 did not hinder its dimerization with IRF3 in HEK-293T cells after SeV infection for 6 h (Fig 7E), suggesting that the neddylation of IRF7 is not required for its dimerization. IRF7 can also dimerize with IRF5 through the DNA-binding domain after viral infection. IRF-5 can function as an *Ifna* activator when present as homodimers or heterodimers with IRF3 but prevent the binding of IRF7 to *Ifna* gene promoters when interacting with IRF7[39,40]. Therefore, we also analyzed how neddylation might affect IRF5/IRF7 interaction. Co-IP analysis revealed that SeV-induced dimerization of IRF7 with IRF5 was significantly enhanced upon K3R mutation (Fig 7F). Thus, the neddylation of IRF7 enhances its transcriptional activity through, at least partially, preventing its dimerization with IRF5, an *Ifna* repressor when interacting with IRF7.

## Discussion

Using mouse models with myeloid deficiency of UBA3 or NEDD8, we report for the first time that neddylation contributes to innate immunity against RNA viruses in mammals (Fig 2). Thus, despite numerous studies indicate that various viruses hijack neddylation for their replication in CRL-dependent or -independent manners [41], our data offer a warning that it’s dangerous to target neddylation for the prevention or treatment of virus infection diseases. Furthermore, MLN4924 has been proved to be effective in many diseases and is in clinical trials for the treatment of malignancies [42–47]. It is pivotal to prevent viral infection in the clinical application of MLN4924 or other neddylation inhibitors. Neddylation is essential for RNA virus-triggered IFN-α expression or *Ifna* promoter activation in all the cell types tested. It also promotes IFN-β expression in mDCs and MEFs and *Ifnb1* promoter activation in HEK-293T cells although such a role is much blunted in macrophages (Figs 1,3 and S2). Despite of the defective type I IFN production, RNA virus-triggered IRF7 expression in macrophages was not hampered upon neddylation blockade (S6A–S6C Fig). A previous study has revealed that a Cullin 1-based ubiquitin E3 ligase mediates ubiquitin-dependent protein turnover of IRF7 before and after viral infection in most cell types [48]. Thus, it is possible that neddylation blockade enhances the stability of IRF7, which makes up for the compromised *Irf7* gene transcription. Indeed, quantitative RT-PCR revealed that the upregulation of *Irf7* in BMDMs after SeV infection for 6 h or 8 h was hampered upon NEDD8 deficiency (S10A Fig). On the other hand, the stability of IRF7 in BMDMs was enhanced in the absence of NEDD8 (S10B Fig).

Furthermore, we have identified mammalian IRF7 as a neddylation substrate. Lys398, Lys400, and Lys406 are key sites for the neddylation of murine IRF7 (Fig 5B and 5C). These key sites are equivalent to Lys444, Lys446, and Lys452 in human IRF7. There’s a report that Lys444, Lys446, and Lys452 are critical for the activation of human IRF7 as key sites for K63-linked ubiquitination [49]. However, K3R mutation did not abolish K63-linked ubiquitination of murine IRF7 in our hands (S11 Fig), suggesting the neddylation of mammalian IRF7 is independent of its K63-linked ubiquitination. Intriguingly, the covalent attachment of NEDD8 to IRF7 seems to promote the formation of an upshifted band at about 60 kDa (Figs 4E and 7D), which can be recognized by an antibody against IRF7, but not NEDD8 (Fig 4E). The composition of this band remains unknown. As K3R mutation fails to hinder SeV-induced phosphorylation of murine IRF7 at Ser437/438 (Fig 7B) and its dimerization with IRF3 (Fig 7D), it is reasonable to propose that certain post translational modification(s) of IRF7 other than phosphorylation depend on its neddylation. The relationship between neddylation and other post translational modifications still needs further study.

Importantly, the covalent attachment of NEDD8 to IRF7 is essential for its transcriptional activity (Fig 5). This finding can explain the abolished IFN-α induction upon neddylation inhibition in all the cell types tested and the diminished IFN-β induction in mDCs, MEFs, and HEK-293T cells after RNA virus infection (Figs 1,3 and S2).IRF7 should also contribute to IFN-β induction in macrophages at early phase of RNA virus infection [13]. Indeed, we observed partially reduced IFN-β expression in NEDD8-deficient or MLN4924-pretreated BMDMs at early phase (4h) of SeV infection (Figs 1F, 1H and 3B). However, no reduction of RNA virus-triggered IFN-β expression was observed in the absence of UBA3 (Figs 1B, 1C and 3A). Similarly, the effects of UBA3 deficiency on SeV-triggered IRF3 phosphorylation and degradation are attenuated, as compared to NEDD8 deficiency or MLN4924 pretreatment (S6A–S6C Fig). Attenuated effects of conditional*Uba3* deletion, as compared to conditional *Nedd8* deletion, were also observed in neonatal livers [37]. It is possible a yet unknown neddylation E1 exists in hepatocytes and macrophages, which can play certain redundant role(s) with UBA3 and can also be inhibited by MLN4924. Another possibility is that UBA3 has certain neddylation-independent role(s) which partially compensate the effects of neddylation blockade. Nevertheless, RNA virus-triggered IFN-α expression in macrophages is significantly reduced upon UBA3 deficiency (Figs 1B, 1C and 3A). Other antiviral/inflammatory genes under the control of IRF7 might also be affected by neddylation. Future studies are required to address these issues.

The underlying mechanisms by which neddylation promotes the transcriptional activity of mammalian IRF7 are of interest. Unexpectedly, we have found that the neddylation of mammalian IRF7 facilitates its nuclear translocation (Fig 6A and 6B) and prevents its dimerization with IRF5 (Fig 7E), an *Ifna* repressor when interacting with IRF7. In line with these observations, we have disclosed that IRF7 neddylation enhances its interaction with nuclear transcriptional co-activator CBP and facilitates its binding to *Ifna* gene promoters within the chromatin (Fig 6C and 6D). In addition, the neddylation of IRF7 might render it to interact with component(s) of the nuclear transcriptional machinery with domains (CUBAN, UIM, UBA [50,51]) that could bind to NEDD8, which might also affect its transcriptional activity.

The neddylation of zebrafish IRF3 was also detected under the conditions of co-overexpression with NEDD8 in HEK-293T cells [28]. However, our data have excluded the neddylation modification of mammalian IRF3 (Fig 4A). Instead, we have observed SeV-induced IRF3 degradation is impaired upon neddylation blockade in macrophages, which is associated with enhanced IRF3 phosphorylation at Ser396, although such effects are marginal in the case of UBA3 deficiency (S6A–S6C Fig). In line with a previous report [26], these effects of neddylation inhibition diminished upon Cullin 1 knockdown (S12 Fig). Thus, neddylation promotes SeV-induced IRF3 degradation through a Cullin 1-based ubiquitin ligase and thereby inhibits SeV-induced IRF3 phosphorylation. However, the regulation of RNA virus-induced IRF3 activation by neddylation may be very complicated. Because the neddylation of IRF7 promotes its nuclear translocation without enhancing its dimerization with IRF3 (Figs 6A, 6B and 7D), it is highly possible that the nuclear translocation of IRF3/IRF7 heterodimers is also facilitated by IRF7 neddylation. In this regard, IRF7 neddylation should enhance the transcriptional activity of IRF3/IRF7 heterodimers as well as IRF7 homodimers.

DNA and RNA viruses all activate transcription factors NF-κB, IRF3, and IRF7 by the same kinases in a given cell context, although through different upstream pattern recognition receptor pathways [1,3]. Intriguingly, HSV-1 only shows modest effects on IRF7 neddylation whereas SeV significantly enhances IRF7 neddylation in a time-dependent manner (Fig 4D). Both viruses exhibit marginal effects on Cullin 1 neddylation but might enhance global neddylation since more neddylated proteins were detected by histidine pulldown after viral infection (Fig 4D). Thus, it is possible that certain component(s) in RNA viruses activate the NEDD8 E3(s) for IRF7.Furthermore, HSV-1-induced IRF7 expression in macrophages was very weak while potent IRF7 induction occurred after SeV infection, even though HSV-1 induced more potent IRF3 phosphorylation at Ser396 (S4 Fig). Thus, it is reasonable to propose that, at least in macrophages, IRF7 neddylation plays a more important role in RNA virus infection than in DNA virus infection, although it might also contribute to DNA virus-induced type I IFN production.

As for NF-κB pathway, at the dose that both SeV and HSV-1 potently induced NF-κB p65 phosphorylation at Ser536 in macrophages, only HSV-1 induced efficient IκBα degradation (S4 Fig, Last lane). Indeed, we previously observed efficient NF-κB p65 nuclear translocation after HSV-1 infection [24], whereas SeV-induced NF-κB p65 nuclear translocation was partial (S7 Fig). Consistently, NF-κB inhibitor JSH-23 significantly dampens HSV-1-induced early phase IFN-β expression and the inhibitory effects of neddylation blockade on early phase IFN-β expression diminishes in the presence of NF-κB inhibitor JSH-23 [24]. On the other hand, NF-κB has been reported to contribute to IFN-β production at early phase of RNA virus infection only when IRF3 activation is weak [25]. Since SeV-triggered IRF3 activation in macrophages is potent and neddylation blockade always leads to enhanced IRF3 phosphorylation at Ser396 (S6A–S6C Fig), it is reasonable to propose the role of NF-κB in SeV-triggered IFN-β production in macrophages is marginal upon neddylation blockade. Future studies are required to address the interplay between these pathways.

## Materials and methods

### Ethics statement

All animal work in this study was approved by the Institutional animal care and use committee of Beijing Institute of Basic Medical Sciences (Permit number: AMMS2015-0119), and was performed in strict accordance with the Guide for the Care and Use of Laboratory Animals in Research of the People’s Republic of China. All efforts were made to minimize suffering.

### Mice

Mice homozygous for a *Uba3* conditional allele (*Uba3*^F/F^) or a *Nedd8* conditional allele (*Nedd8*^F/F^) on a C57BL/6 background have been described [24,37,52]. *Uba3*^F/F; Lyz2-Cre^ (named as *Uba3*^ΔMye^) and *Nedd8*^F/F; Lyz2-Cre^ (named as *Nedd8*^ΔMye^) mice of C57 BL/6 strain were then generated. All mice were bred and maintained under specific pathogen-free conditions and used between 8 to 9 weeks of age.

### Virus

Influenza A/Puerto Rico/8/1934 H1N1 virus was provide by State Key Laboratory of Pathogen and Biosecurity, Beijing Institute of Microbiology and Epidemiology. SeV and HSV-1 were kindly provided by Dr. Zhengfan Jiang (Peking University). SeV and H1N1 were propagated in embryonated chicken eggs and HSV-1 was propagated in HeLa cells. For cell-based assays, cells were infected with SeV (1 MOI or serially diluted), HSV-1 (1 MOI or serially diluted), or H1N1 (5 MOI) for the indicated time periods. For *in vivo* infection, each mouse was anaesthetized and then challenged intranasally with 500 PFU H1N1 in 40 μl virus collection medium.

### Plasmids and siRNAs

Mammalian expression vectors encoding FLAG-Myc-tagged murine IRF7 (Cat. No. MR225814), FLAG-tagged human IRF3 (Cat. No. HG12007), FLAG-tagged murine CBP (Cat. No. 32908), and FLAG-tagged human IRF5 (Cat. No. CH890176) were obtained from Origene (Rockville, MD, USA), Sino Biological Inc. (Beijing, China), Addgene (Watertown, MA, USA) [53], and Vigenebio (Jinan, Shandong, China), respectively. Prokaryotic expression vector encoding His-tagged murine IRF7 was generated by cloning a synthetic gene with optimized codons into pET-28a vector and confirmed by DNA sequencing. The mutants of IRF7 were constructed using Fast Mutagenesis System kit (Cat. No. FM111, TransGen Biotech, Beijing, China). His-tagged NEDD8 plasmid and HA-tagged K63onlyUb plasmid have been described previously [37,54]. pEZX-PG04 reporter plasmids carrying *Gaussia* Luciferase (GLuc) gene driven by *Ifn-α4* promoter (-1441~ +29, Cat. No. MPRM41250) or *Ifn-α6* promoter (-1450~ +1, Cat. No. MPRM52342) were ordered from Genecopia (Rockville, MD, USA). *Ifnb1* luciferase reporter and pRL control vector were kindly provided by Dr. Hui Zhong (Beijing Institute of Biotechnology) and has been described previously [55]. Murine UBA3 siRNA (CCTGACATCTAGAGTATAT), human UBA3 siRNA (CACAGACTGTACTATTCAATT), Cullin 1 siRNA (GCCATTGAATAAACAGGTA), and the non-targeting control (NC) siRNA were purchased from Shanghai Gene Pharma (Shanghai, China).

### Cell culture and transfection

Bone marrow-derived macrophages and myeloid dendritic cells were obtained by culturing the nonadherent bone marrow cells in RPMI-1640 medium containing 15% (v/v) FBS, 2 mM L-glutamine, 100 U/ml penicillin, 100 mg/ml streptomycin, and 50 mM 2-ME with 100 ng/ml M-CSF (Cat. No. 216-MC-025, R&D Systems, Minneapolis, MN, USA) or 20 ng/ml GM-CSF (Cat. No. 500-P65) and 10 ng/ml IL-4 (Cat. No. 214–14, Peprotech, Rocky Hill, NJ, USA) for 7 days, respectively. The survival of BMDMs was monitored with ATPlite 1step Luminescence Assay System (Cat. No. 50-904-9883, PerkinElmer, Waltham, MA, USA). Cell lines and MEFs were cultured in DMEM complete medium. Plasmids and siRNAs were transfected with Lipofectamine 2000 (Cat. No. 52887) and Lipofectamine RNAiMAX (Cat. No. 13778075, Invitrogen, Carlsbad, CA, USA), respectively, according to the manufacture’s protocols.

### ELISA

The levels of type I IFNs in the serum and cell culture supernatants were determined using ELISA kits according to the manufacturers’ protocols. IFN-α ELISA kit (Cat. No. 42120–1) was from PBL Assay Science (Piscataway, NJ, USA). IFN-β ELISA kit (Cat. No. 439407) was from Biolegend (San Diego, CA, USA).

### Histology and immunohistochemistry

Lung tissues were removed from mice and fixed in 10% buffered formalin for at least 24 h, dehydrated, and infiltrated with paraffin. 5 μm paraffin sections were then prepared and stained with hematoxylin and eosin (H & E) for bright field microscopy. IHC was performed using standard protocols with citrate buffer (pH 6.0) pretreatment. Briefly, formaldehyde-fixed and paraffin-embedded lung sections were incubated with an antibody against F4/80 (Cat. No. sc-52664, Santa Cruz Biotechnology, Santa Cruz, CA, USA) at 4°C overnight and then with horseradish peroxidase-conjugated secondary antibodies at 37°C for 30 min. The sections were finally incubated with diaminobenzidine and counterstained with hematoxylin for detection.

### Quantitative RT-PCR

Total RNA was extracted with Trizol reagent (Life Technologies, CA, USA). A total amount of 1 μg RNA per sample was used as input material for sample preparations. First-strand synthesis was performed with Oligo dT primers and reverse transcription was performed with M-MLV reverse transcriptase (Cat. No. B24403, Bimake, Shanghai, China). The amplification was performed with SYBR Green Realtime PCR Mix (Cat. No. QPK-201, TOYOBO Life Sciences, Tokyo, Japan) on a CFX96 Real-Time System (BIO-RAD, Hercules, CA, USA). Relative expression of target genes was normalized to the *Gapdh* internal control (2^-ΔΔCt^ method). The primer sequences were listed in S1 Table.

### IB and Co-IP

IB and Co-IP were carried out as described previously [37,56]. Cells were harvested in RIPA buffer (50mM Tris-HCl, pH 7.5, 1% NP40, 0.35% DOC, 150mM NaCl, 1mM EDTA, 1mM EGTA, supplemented with protease and phosphatase inhibitor cocktails) or IP lysis buffer (10mM Tris-HCl, pH 7.5, 2mM EDTA, 1% NP40, 150mM NaCl, supplemented with protease and phosphates inhibitor cocktail). Nuclear cytoplasmic fractionation was performed with a commercial kit (Cat. No. P0027, Beyotime Biotechnology, Shanghai, China) according to the manufacturer’s protocol. Primary antibodies used are listed in S2 Table.

### *In vivo* neddylation assay with histidine pulldown

Histidine pulldown assays were performed as described previously [37,57]. In brief, the transfected cells were lysed in buffer A (6 M guanidine-HCl, 0.1 M Na_2_HPO4/NaH_2_PO4, 10 mM imidazole, pH 8.0). After sonication, lysates were centrifuged at 10,000 g for 30 min at 4°C, and the supernatant fractions were incubated with nickel-nitrilotriacetic acid (Ni-NTA) resin (Qiagen, Hilden, Germany) overnight at room temperature. Histidine pulldown products were washed sequentially once in buffer A, twice in buffer A and buffer TI (25 mM Tris-Cl and 20 mM imidazole, pH 6.8) mixture (buffer A: buffer TI = 1:3), and once in buffer TI. Precipitates were separated by SDS-PAGE for IB analysis.

### *In vivo* neddylation assay with IP

Cells were solubilized in modified lysis buffer (50 mM Tris-Cl, pH 7.4, 150 mM NaCl, 10% glycerol, 1 mM EDTA, 1 mM EGTA, 1% SDS, 1 mM Na_3_VO_4_, 1 mM DTT, and 10 mM NaF) supplemented with protease inhibitor cocktail, as previously described [37]. The cell lysates were incubated at 60°C for 10 min, followed by 10 times dilution with modified lysis buffer without SDS. After sonication, samples were incubated at 4°C for 1 h with rotation, followed by centrifugation (14,000 rpm) for 30 min at 4°C. After the protein concentration was determined by the Bradford assay, appropriate amounts (0.5–1.5 mg) of protein were used for IP. Immunoprecipitated proteins were washed with washing buffer (50 mM Tris-Cl, pH 7.4, 500 mM NaCl, 10% glycerol, 1 mM EDTA, 1 mM EGTA, 0.1% SDS, 1 mM DTT, and 10 mM NaF) three times, boiled in SDS sample buffer, and separated on SDS-PAGE. To detect neddylation sites, smear bands were cut and subjected to mass spectrometry after silver staining. The mass spectrometry proteomics data have been deposited to the ProteomeXchange Consortium via the PRIDE [58] partner repository with the dataset identifier PXD024052 (http://www.ebi.ac.uk/pride/archive/projects/PXD024052).

### *In vitro* neddylation assay

Bacterially expressed His-tagged murine IRF7 WT and mutants were purified with Ni-NTA resin (Qiagen). *In vitro* neddylation of His-IRF7 (200 ng in each sample, 37°C, 1 h) was performed with a commercial kit (Cat. No. BML-UW0590, Enzo Life Sciences, Farmingdale, NY, USA), according to the manufacturer’s instructions. The neddylation of His-IRF7 was analyzed by IB.

### Dual-reporter luciferase assay

HEK-293T cultured in 24-well plates were transfected with 50 ng pEZX-PG04 reporter plasmids carrying GLuc gene driven by *Ifna4* or *Ifna6* promoter. The reporter vector also contains Secreted Alkaline Phosphatase (SEAP) gene under the control of constitutively active CMV promoter, which allows normalization of Gluc signal for greater accuracy. Both Gluc and SEAP are secreted reporter proteins, permitting detection without cell lysis. Twenty-four hours after transfection, cells were infected with SeV for 24 or 36 h or left untreated. *Ifna4* and *Ifna6* gene promoter activity was then measured with the Secrete-Pair Dual Luminescence Assay Kit (Cat. No. LF032, Genecopia) according to the manufacturer’s protocol.

HEK-293T cultured in 24-well plates were transfected with 50 ng *Ifnb1* luciferase reporter carrying firefly luciferase gene driven by *Ifnb1* promoter and 10 ng pRL control vector carrying Renilla luciferase gene under the control of constitutively active SV40 promoter. 24 h after transfection, cells were infected with SeV for 24 h or left untreated. Then cells were lysed and the luciferase activity was measured with the Dual Luciferase Reporter Assay kit (Promega).

### ChIP

A SimpleChIP® Plus Sonication ChIP kit (Cat. No. 56383, Cell Signaling Technology, Danvers, MA, USA) was used for this purpose. Briefly, single-cell suspensions were harvested and fixed for 10 min at room temperature with 1% formaldehyde. After shearing the genomic DNA by sonication, Protein G Magnetic Beads and mouse anti-Myc-tag antibody (Cat. No. 598, MBL International, Woburn, MA, USA) or normal mouse IgG were added into each sample, followed by incubation at 4°C overnight with rotation. After reversal of protein-DNA cross-links, the DNA was purified using DNA purification spin columns and subjected to quantitative PCR. The primer sequences were listed in S1 Table.

### Statistical analysis

All experiments were repeated at least three times with consistent results. All statistical analyses were performed with GraphPad Prism software version 6.01 (GraphPad Software Inc., San Diego, CA, USA). Differences among experimental groups were assessed using analysis of variance (ANOVA) or two-tailed Student’s *t* test. Kaplan-Meier curves of overall survival were compared using the log-rank test. *p* values less than 0.05 were considered statistically significant.

## Supporting information

S1 Fig Effects of myeloid neddylation blockade on the frequencies of macrophages *in vivo*. Flow cytometric analysis of F4/80+CD11b+macrophage populations in peripheral blood (PB), bone marrow (BM), spleen (SP), and peritoneal cavity (PC) of *Uba3*^ΔMye^ and *Nedd8*^ΔMye^ mice and their control littermates (*n* = 6 per group).
(TIF)

S2 Fig Effects of neddylation blockade on SeV-triggered type I IFN production in MEFs. (A) IB analysis to confirm the efficiency of MLN4924 pretreatment (0.5 μM, 3 h) in MEFs. (B) After MLN4924 pretreatment (0.5 μM, 3 h), MEFs were infected with SeV for the indicated time periods. Then the supernatants were subjected to ELISA. Quantitative data are shown as Mean ± SD (*n* = 3 per group). ***p< 0.001.
(TIF)

S3 Fig *In vivo* neddylation assay of exogenous murine IRF7 with IP. HEK-293T cells were transfected with the indicated mammalian expression vectors. Twenty-four hours later, possible neddylation of exogenous murine IRF7 was examined by IB analysis with the indicated antibodies after IP under partially denaturing conditions with an antibody against GFP.
(TIF)

S4 Fig Different effects of SeV and HSV-1 on innate immune signaling. WT BMDMs were infected with different doses of the indicated viruses for 6 h. Cell lysates were then harvested and subjected to IB analysis with the indicated antibodies. P-p65, phosphorylated p65 at Ser536; P-IRF3, phosphorylated IRF3 at Ser396; ns, non-specific band.
(TIF)

S5 Fig The interaction between IRF7 and Ubc12. IB analysis of the interaction between endogenous IRF7 and endogenous Ubc12 in SeV-infected Raw264.7 after IP with an anti-IRF7 antibody (*Left*) or an anti-Ubc12 antibody (*Right*). Control antibody: rabbit IgG; IgG HC, IgG heavy chain; ns, non-specific.
(TIF)

S6 Fig Neddylation does not promote RNA virus-induced IRF7 expression. (A-C) After BMDMs were cultured from the indicated mouse models (A-B) or WT BMDMs were pretreated with 0.1 μM MLN4924 for 3 h (C), the cells were infected with SeV for the indicated time periods. Cell lysates were then harvested and subjected to IB analysis with the indicated antibodies (*Top*). P-TBK1, phosphorylated TBK1 at Ser172; P-p65, phosphorylated p65 at Ser536; P-IκBα, phosphorylated IκBα at Ser32; P-IRF3, phosphorylated IRF3 at Ser396; ns, non-specific band. The density of the indicated bands was quantified by scanning densitometry and normalized to β-actin (*Bottom*). Quantitative data are shown as Mean ± SD (*n* = 3 per group). *p< 0.05; **p< 0.01; ***p< 0.001; NS, not significant.
(TIF)

S7 Fig SeV-triggered NF-κB p65 nuclear translocation in the presence and absence of UBA3. Six hours after BMDMs from *Uba3*^F/F^ and *Uba3*^ΔMye^ mice were infected with SeV or left uninfected, the nuclear translocation of NF-κB was examined by indirect immunofluorescence analysis with an antibody against p65 (Scale bar, 10 μm).
(TIF)

S8 Fig Effects of lysine to arginine mutation on IRF7 neddylation. HEK-293T cells were transfected with mammalian expression vectors encoding His-NEDD8 and FLAG-Myc-tagged murine IRF7 WT or mutants. Twenty-four hours later, neddylation of exogenous murine IRF7 was examined by IB analysis with the indicated antibodies after histidine pulldown under fully denaturing conditions.
(TIF)

S9 Fig Possible neddylated peptides of murine IRF7 as revealed by mass spectrometry analysis. (PDF)

S10 Fig Effects of NEDD8 deficiency on SeV-induced *Irf7* mRNA upregulation and IRF7 stability. (A) BMDMs from *Nedd8*^F/F^ and *Nedd8*^ΔMye^ mice were infected with SeV for the indicated time periods. Then cells were subjected to quantitative RT-PCR analysis. Data are shown as Mean ± SD (*n* = 4 per group). *p< 0.05; **p< 0.01; NS, not significant. (B) After BMDMs from *Nedd8*^F/F^ and *Nedd8*^ΔMye^ mice were infected with or without SeV for 6 h, the cells were treated with 10 μg/mL cycloheximide (CHX) for various periods of time. Then, the half-life of IRF7 was analyzed by IB. ns, non-specific band. Densitometric readings are shown for IRF7 and normalized to β-actin.
(TIF)

S11 Fig Effects of neddylation on K63-linked ubiquitination of murine IRF7. HEK-293T cells were transfected with mammalian expression vectors encoding HA-K63onlyUb and FLAG-Myc-tagged murine IRF7 WT or K3R mutant. After 12 h, cells were treated with 0.5μM MLN4924 for another 24 h or left untreated. The modification of exogenous murine IRF7 was then examined by IB analysis with the indicated antibodies after IP with an antibody against Myc-tag. IgG HC, IgG heavy chain.
(TIF)

S12 Fig Effects of UBA3 and Cullin 1 on SeV-induced IRF3 phosphorylation in MEFs. Forty-eight hours after MEFs were transfected with the indicated siRNAs, cells were infected with SeV for the indicated time periods. Cell lysates were then harvested and subjected to immunoblotting analysis with the indicated antibodies. P-TBK1, phosphorylated TBK1 at Ser172; P-IRF3, phosphorylated IRF3 at Ser396; ns, non-specific band.
(TIF)

S1 Table Primers for quantitative RT-PCR. (PDF)

S2 Table Primary antibodies used in this study. (PDF)

## References

[1] Akira S, Uematsu S, Takeuchi O. Pathogen recognition and innate immunity. Cell. 2006;124:783–801. doi: 10.1016/j.cell.2006.02.015 16497588

[2] Loo YM, Gale M Jr. Immune signaling by RIG-I-like receptors. Immunity. 2011;34:680–692. doi: 10.1016/j.immuni.2011.05.003 21616437 PMC3177755

[3] Sadler AJ, Williams BR. Interferon-inducible antiviral effectors. Nat Rev Immunol. 2008;8:559–568. doi: 10.1038/nri2314 18575461 PMC2522268

[4] Wathelet MG, Lin CH, Parekh BS, Ronco LV, Howley PM, Maniatis T. Virus infection induces the assembly of coordinately activated transcription factors on the IFN-beta enhancer in vivo. Mol Cell. 1998;1:507–518. doi: 10.1016/s1097-2765(00)80051-9 9660935

[5] Lin R, Heylbroeck C, Pitha PM, Hiscott J. Virus-dependent phosphorylation of the IRF-3 transcriptionfactor regulates nuclear translocation, transactivation potential, and proteasome-mediated degradation. Mol Cell Biol. 1998;18:2986–2996. doi: 10.1128/MCB.18.5.2986 9566918 PMC110678

[6] Yoneyama M, Suhara W, Fukuhara Y, Fukuda M, Nishida E, Fujita T. Direct triggering of the type I interferon system by virus infection: activation of a transcription factor complex containing IRF-3 and CBP/p300. EMBO J. 1998;17:1087–1095. doi: 10.1093/emboj/17.4.1087 9463386 PMC1170457

[7] Sato M, Suemori H, Hata N, Asagiri M, Ogasawara K, Nakao K, et al. Distinct and essential roles of transcription factors IRF-3 and IRF-7 in response to viruses for IFN-alpha/beta gene induction. Immunity. 2000;13:539–548. doi: 10.1016/s1074-7613(00)00053-4 11070172

[8] Marie I, Durbin JE, Levy DE. Differential viral induction of distinct interferon-alpha genes by positive feedback through interferon regulatory factor-7. EMBO J. 1998;17: 6660–6669. doi: 10.1093/emboj/17.22.6660 9822609 PMC1171011

[9] Hiscott J. Convergence of the NF-kappaB and IRF pathways in the regulation of the innate antiviral response. Cytokine Growth Factor Rev. 2007;18:483–490. doi: 10.1016/j.cytogfr.2007.06.002 17706453

[10] Hatesuer B, Hoang HTT, Riese P, Trittel S, Gerhauser I, Elbahesh H, et al. Deletion of *Irf3* and *Irf7* genes in mice results in altered interferon pathway activation and granulocyte-dominated inflammatory responses to influenza A infection. J Innate Immun. 2017;9:145–161. doi: 10.1159/000450705 27811478 PMC6738875

[11] Honda K, Yanai H, Negishi H, Asagiri M, Sato M, Mizutani T, et al. IRF-7 is the master regulator of type-I interferon-dependent immune responses. Nature. 2005;434:772–777. doi: 10.1038/nature03464 15800576

[12] Bhalla N, Gardner CL, Downs SN, Dunn M, Sun C, Klimstra WB. Macromolecular synthesis shutoff resistance by myeloid cells is critical to IRF7-dependent systemic interferon alpha/beta induction after alphavirus infection. J Virol. 2019;93:e00872–19. doi: 10.1128/JVI.00872-19 31578290 PMC6880179

[13] Daffis S, Suthar MS, Szretter KJ, Gale M Jr, Diamond MS. Induction of IFN-β and the innate antiviral response in myeloid cells occurs through an IPS-1-dependent signal that does not require IRF3 and IRF7. PLoS Pathog. 2009;5:e1000607. doi: 10.1371/journal.ppat.1000607 19798431 PMC2747008

[14] Williams DW, Askew LC, Jones E, Clements JE. CCR2 signaling selectively regulates IFN-α: role of β-arrestin in IFNAR1 internalization. J Immunol. 2019;202:105–118. doi: 10.4049/jimmunol.1800598 30504423 PMC6310093

[15] Spencer E, Jiang J, Chen ZJ. Signal-induced ubiquitination of IkappaBalpha by the F-box protein Slimb/beta-TrCP. Genes Dev. 1999;13:284–294. doi: 10.1101/gad.13.3.284 9990853 PMC316434

[16] Winston JT, Strack P, Beer-Romero P, Chu CY, Elledge SJ, Harper JW. The SCFbeta-TRCP-ubiquitin ligase complex associates specifically with phosphorylated destruction motifs in IkappaBalpha and beta-catenin and stimulates IkappaBalpha ubiquitination in vitro. Genes Dev. 1999;13:270–283. doi: 10.1101/gad.13.3.270 9990852 PMC316433

[17] Yaron A, Hatzubai A, Davis M, Lavon I, Amit S, Manning AM, et al. Identification of the receptor component of the IkappaBalpha-ubiquitin ligase. Nature. 1998;398:590–594. doi: 10.1038/25159 9859996

[18] Soffer RL. Post-translational modification of proteins catalyzed by aminoacyl-tRNA-protein transferases. Mol Cell Biochem. 1973;2:3–14. doi: 10.1007/BF01738673 4587539

[19] Bohnsack RN, Haas AL. Conservation in the mechanism of Nedd8 activation by the human AppBp1-Uba3 heterodimer. J Biol Chem. 2003;278:26823–26830. doi: 10.1074/jbc.M303177200 12740388

[20] Xirodima DP, Saville MK, Bourdon JC, Hay RT, Lane DP. Mdm2-mediated NEDD8 conjugation of p53 inhibits its transcriptional activity. Cell. 2004;118:83–97. doi: 10.1016/j.cell.2004.06.016 15242646

[21] Malik-Chaudhry HK, Gaieb Z, Saavedra A, Reyes M, Kung R, Le F, et al. Dissectingdistinct roles of NEDDylation E1 ligase heterodimer APPBP1 and UBA3 reveals potential evolution process for activation of ubiquitin-related pathways. Sci Rep. 2018;8:10108. doi: 10.1038/s41598-018-28214-2 29973603 PMC6031683

[22] Walden H, Podgorski MS, Huang DT, Miller DW, Howard RJ, Minor DL, Jr., et al. The structure of the APPBP1-UBA3-NEDD8-ATP complex reveals the basis for selective ubiquitin-like protein activation by an E1. Mol Cell. 2003;12:1427–1437. doi: 10.1016/s1097-2765(03)00452-0 14690597

[23] Yashiroda H, Tanaka K. But1 and But2 proteins bind to Uba3, a catalytic subunit of E1 for neddylation, in fission yeast. Biochem Biophys Res Commun. 2003;311:691–695. doi: 10.1016/j.bbrc.2003.10.058 14623327

[24] Zhang X, Ye Z, Pei Y, Qiu G, Wang Q, Xu Y, et al. Neddylation is required for herpes simplex virus type I (HSV-1)-induced early phase interferon-beta production. Cell Mol Immunol. 2016;13:578–583. doi: 10.1038/cmi.2015.35 27593482 PMC5037273

[25] Wang J, Basagoudanavar SH, Wang X, Hopewell E, Albrecht R, Garcia-Sastre A, et al. NF-kappa B RelA subunit is crucial for early IFN-beta expression and resistance to RNA virus replication. J Immunol. 2010;185:1720–1729. doi: 10.4049/jimmunol.1000114 20610653 PMC2910841

[26] Bibeau-Poirier A, Gravel SP, Clement JF, Rolland S, Rodier G, Coulombe P, et al. Involvement of the IkappaB kinase (IKK)-related kinases tank-binding kinase 1/IKKi and cullin-based ubiquitin ligases in IFN regulatory factor-3 degradation. J Immunol. 2006;177:5059–5067. doi: 10.4049/jimmunol.177.8.5059 17015689

[27] Song H, Huai W, Yu Z, Wang W, Zhao J, Zhang L, et al. MLN4924, a first-in-class NEDD8-activating enzyme inhibitor, attenuates IFN-β production. J Immunol. 2016;196:3117–3123. doi: 10.4049/jimmunol.1501752 26895833

[28] Yu G, Liu X, Tang J, Xu C, Ouyang G, Xiao W. Neddylation facilitates the antiviral response in zebrafish. Front Immunol. 2019;10:1432. doi: 10.3389/fimmu.2019.01432 31293590 PMC6603152

[29] Yao Q, Cui J, Wang J, Li T, Wan X, Luo T, et al. Structural mechanism of ubiquitin and NEDD8 deamidation catalyzed by bacterial effectors that induce macrophage-specific apoptosis. Proc Natl Acad Sci USA. 2012;109:20395–20400. doi: 10.1073/pnas.1210831109 23175788 PMC3528514

[30] Kumagai Y, Takeuchi O, Kato H, Kumar H, Matsui K, Morii E, et al. Alveolar macrophages are the primary interferon-alpha producer in pulmonary infection with RNA viruses. Immunity. 2007;27:240–252. doi: 10.1016/j.immuni.2007.07.013 17723216

[31] Kumar PA, Hu Y, Yamamoto Y, Hoe NB, Wei TS, Mu D, et al. Distal airway stem cells yield alveoli in vitro and during lung regeneration following H1N1 influenza infection. Cell. 2011;147:525–538. doi: 10.1016/j.cell.2011.10.001 22036562 PMC4040224

[32] Marie I, Smith E, Prakash A, Levy DE. Phosphorylation-induced dimerization of interferon regulatory factor 7 unmasks DNA binding and a bipartite transactivation domain. Mol Cell Biol. 2000;20:8803–8814. doi: 10.1128/MCB.20.23.8803-8814.2000 11073981 PMC86519

[33] Hjerpe R, Thomas Y, Chen J, Zemla A, Curran S, Shpiro N, et al. Changes in the ratio of free NEDD8 to ubiquitin triggers NEDDylation by ubiquitin enzymes. Biochem J. 2012;441:927–936. doi: 10.1042/BJ20111671 22004789 PMC3280039

[34] Xie P, Peng Z, Chen Y, Li H, Du M, Tan Y, et al. Neddylation of PTEN regulates its nuclear import and promotes tumor development. Cell Res. 2021;31:291–311. doi: 10.1038/s41422-020-00443-z 33299139 PMC8027835

[35] Aoki I, Higuchi M, Gotoh Y. NEDDylation controls the target specificity of E2F1 and apoptosis induction. Oncogene. 2013;32:3954–3964. doi: 10.1038/onc.2012.428 23001041

[36] Zhu T, Wang J, Pei Y, Wang Q, Wu Y, Qiu G, et al. Neddylation controls basal MKK7 kinase activity in breast cancer cells. Oncogene. 2016;35:2624–2633. doi: 10.1038/onc.2015.323 26364603

[37] Zhang X, Zhang YL, Qiu G, Pian L, Guo L, Cao H, et al. Hepatic neddylation targets and stabilizes electron transfer flavoproteins to facilitate fatty acid β-oxidation. Proc Natl Acad Sci U S A. 2020;117:2473–2483. doi: 10.1073/pnas.1910765117 31941714 PMC7007566

[38] Genin P, Lin R, Hiscott J, Civas A. Differential regulation of human interferon A gene expression by interferon regulatory factors 3 and 7. Mol Cell Biol. 2009;29:3435–3450. doi: 10.1128/MCB.01805-08 19349300 PMC2698742

[39] Barnes BJ, Field AE, Pitha-Rowe PM. Virus-induced heterodimer formation between *IRF5* and *IRF7* modulates assembly of the *IFNA* enhanceosome in vivo and transctiptional activity of *IFNA* genes. J Biol Chem. 2003;278:16630–16641. doi: 10.1074/jbc.M212609200 12600985

[40] Ning S, Huye LE, Pagano JS. Interferon regulatory factor 5 represses expression of the Epstein-Barr virus oncoprotein LMP1: Braking of the IRF7/LMP1 regulatory circuit. J Virol. 2005;79:11671–11676. doi: 10.1128/JVI.79.18.11671-11676.2005 16140744 PMC1212628

[41] Zou T, Zhang J. Diverse and pivotal roles of neddylation in metabolism and immunity. FEBS J. 288:3884–921. doi: 10.1111/febs.15584 33025631

[42] Swords RT, Erba HP, DeAngelo DJ, Bixby DL, Altman JK, Maris M, et al. Pevonedistat (MLN4924), a first-in-class NEDD8-activating enzyme inhibitor, in patients with acute myeloid leukaemia and myelodysplastic syndromes: a phase 1 study. Br J Haematol. 2015;169:534–543. doi: 10.1111/bjh.13323 25733005

[43] Shah JJ, Jakubowiak AJ, O’Connor OA, Orlowski RZ, Harvey RD, Smith MR, et al. Phase I study of the novel investigational NEDD8-activating enzyme inhibitor pevonedistat (MLN4924) inpatients with relapsed/refractory multiple myeloma or lymphoma. Clin Cancer Res. 2016;22:34–43 doi: 10.1158/1078-0432.CCR-15-1237 26561559 PMC5694347

[44] Sarantopoulos J, Shapiro GI, Cohen RB, Clark JW, Kauh JS, Weiss GJ, et al. Phase I study of the investigational NEDD8-activating enzyme inhibitor pevonedistat (TAK-924/MLN4924) in patients with advanced solid tumors. Clin Cancer Res. 2016;22:847–857. doi: 10.1158/1078-0432.CCR-15-1338 26423795

[45] Bhatia S, Pavlick AC, Boasberg P, Thompson JA, Mulligan G, Pickard MD, et al. A phase I study of the investigational NEDD8-activating enzyme inhibitor pevonedistat (TAK-924/MLN4924) in patients with metastatic melanoma. Invest New Drugs. 2016;34:439–449. doi: 10.1007/s10637-016-0348-5 27056178 PMC4919369

[46] Swords RT, Watts J, Erba HP, Altman JK, Maris M, Anwer F, et al. Expanded safety analysis of pevonedistat, a first-in-class NEDD8-activating enzyme inhibitor, in patients with acute myeloid leukemia and myelodysplastic syndromes. Blood Cancer J. 2017;7:e520 doi: 10.1038/bcj.2017.1 28157218 PMC5386335

[47] Swords RT, Coutre S, Maris MB, Zeidner JF, Foran JM, Cruz J, et al. Pevonedistat, a first-in-class NEDD8-activating enzyme inhibitor, combined with azacitidine in patients with AML. Blood. 2018;131:1415–1424. doi: 10.1182/blood-2017-09-805895 29348128 PMC5909884

[48] Prakash A, Levy DE. Regulation of IRF7 through cell type-specific protein stability. Biochem Biophys Res Commun. 2006;342:50–56. doi: 10.1016/j.bbrc.2006.01.122 16472772 PMC1647301

[49] Ning S, Campos AD, Darnay BG, Bentz GL, Pagano JS. TRAF6 and the three C-terminal lysine sites on IRF7 are required for its ubiquitination-mediated activation by the tumor necrosis factor receptor family member latent membrane protein 1. Mol Cell Biol. 2008;28:6536–6546. doi: 10.1128/MCB.00785-08 18710948 PMC2577435

[50] Santonico E. Old and new concepts in ubiquitin and NEDD8 recognition. Biomolecules. 2020;10:566. doi: 10.3390/biom10040566 32272761 PMC7226360

[51] Santonico E, Nepravishta R, Mandaliti W, Castagnoli L, Cesareni G, Paci M. CUBAN, a case study of selective binding: structural details of the discrimination between ubiquitin and NEDD8. Int J Mol Sci. 2019;20:1185. doi: 10.3390/ijms20051185 30857167 PMC6429362

[52] Cheng Q, Liu J, Pei Y, Zhang Y, Zhou D, Pan W, et al. Neddylation contributes to CD4+ T cell-mediated protective immunity against blood-stage Plasmodium infection. PLoS Pathog. 2018;14:e1007440. doi: 10.1371/journal.ppat.1007440 30462731 PMC6249024

[53] Zhao X, Sternsdorf T, Bolger TA, Evans RM, Yao TP. Regulation of MEF2 by histone deacetylase 4- and SIRT1 deacetylase-mediated lysine modifications. Mol Cell Biol. 2005;25:8456–8464. doi: 10.1128/MCB.25.19.8456-8464.2005 16166628 PMC1265742

[54] Zhang L, Wang Y, Xiao F, Wang S, Xing G, Li Y, et al. CKIP-1 regulates macrophage proliferation by inhibiting TRAF6-mediated Akt activation. Cell Res. 2014;24:742–761. doi: 10.1038/cr.2014.53 24777252 PMC4042176

[55] He X, Zhu Y, Zhang Y, Geng Y, Gong J, Geng J, et al. RNF34 function in immunity and selective mitophagy by targeting MAVS for autophagic degradation. EMBO J. 38:e100978. doi: 10.15252/embj.2018100978 31304625 PMC6627233

[56] Wang B, Jie Z, Joo D, Ordureau A, Liu P, Gan W, et al. TRAF2 and OTUD7B govern a ubiquitin-dependent switch that regulates mTORC2 signalling. Nature. 2017;545:365–369. doi: 10.1038/nature22344 28489822 PMC5695540

[57] Cao J, Zhao M, Liu J, Zhang X, Pei Y, Wang J, et al. RACK1 promotes self-renewal and chemoresistance of cancer stem cells in human hepatocellular carcinoma through stabilizing Nanog. Theranostics. 2019;9:811–828. doi: 10.7150/thno.29271 30809310 PMC6376462

[58] Deutsch EW, Bandeira N, Sharma V, Perez-Riverol Y, Carver JJ, Kundu DJ, et al. The ProteomeXchange consortium in 2020: enabling ‘big data’ approaches in proteomics, Nucleic Acids Res. 2020;48:D1145–D1152. doi: 10.1093/nar/gkz984 31686107 PMC7145525

