# Supplementary figures and images for: Myeloid neddylation targets IRF7 and promotes host innate immunity against RNA viruses
Source: PLoS Pathog. 2021 Sep 10;17(9):e1009901. doi: 10.1371/journal.ppat.1009901 (PMC8432861; doi:10.1371/journal.ppat.1009901)

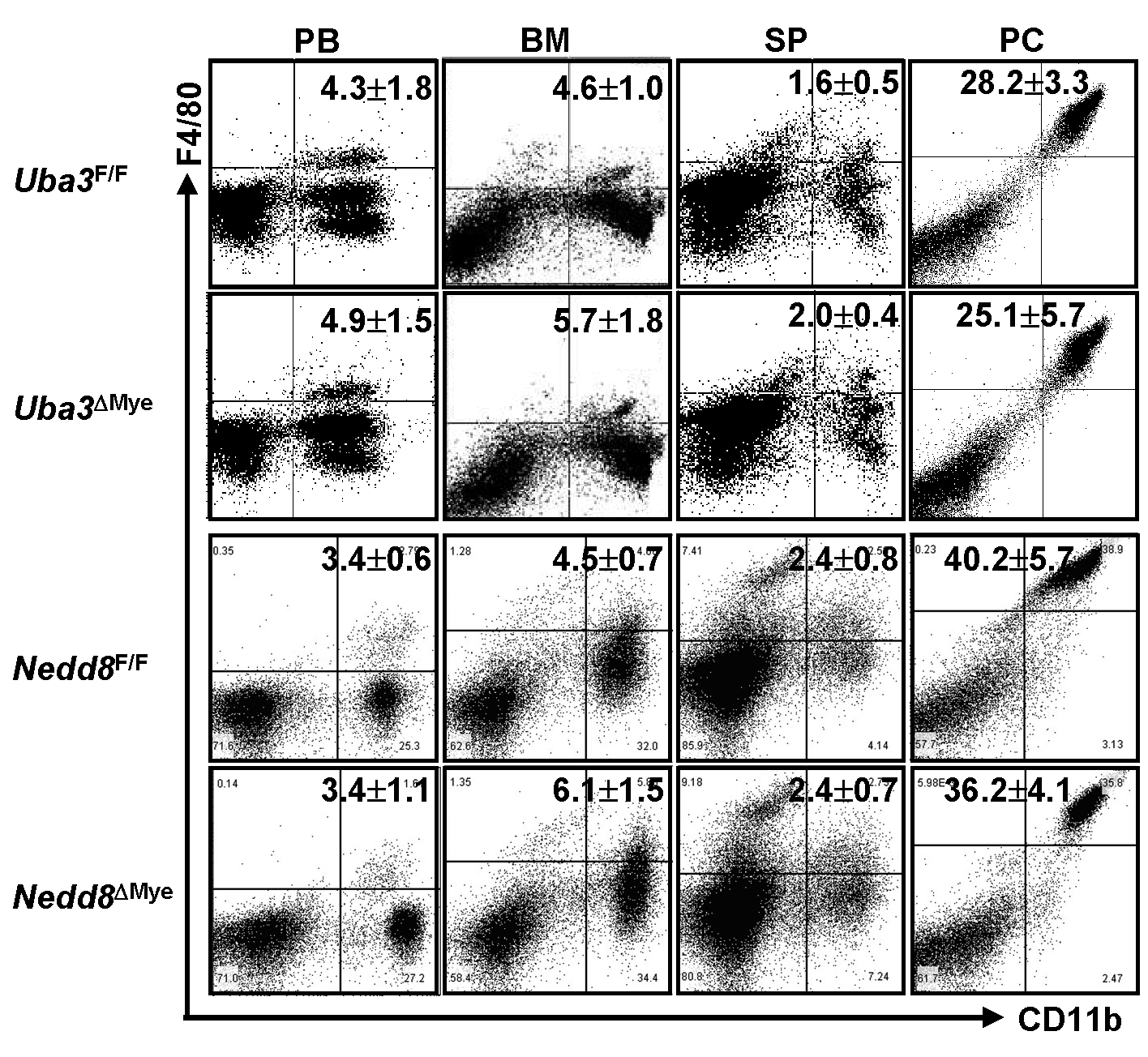

Supplement: S1 Fig — Flow cytometric analysis of F4/80+CD11b+macrophage populations in peripheral blood (PB), bone marrow (BM), spleen (SP), and peritoneal cavity (PC) of Uba3ΔMye and Nedd8ΔMye mice and their control littermates (n = 6 per group). (TIF) [file ppat.1009901.s001.tif]

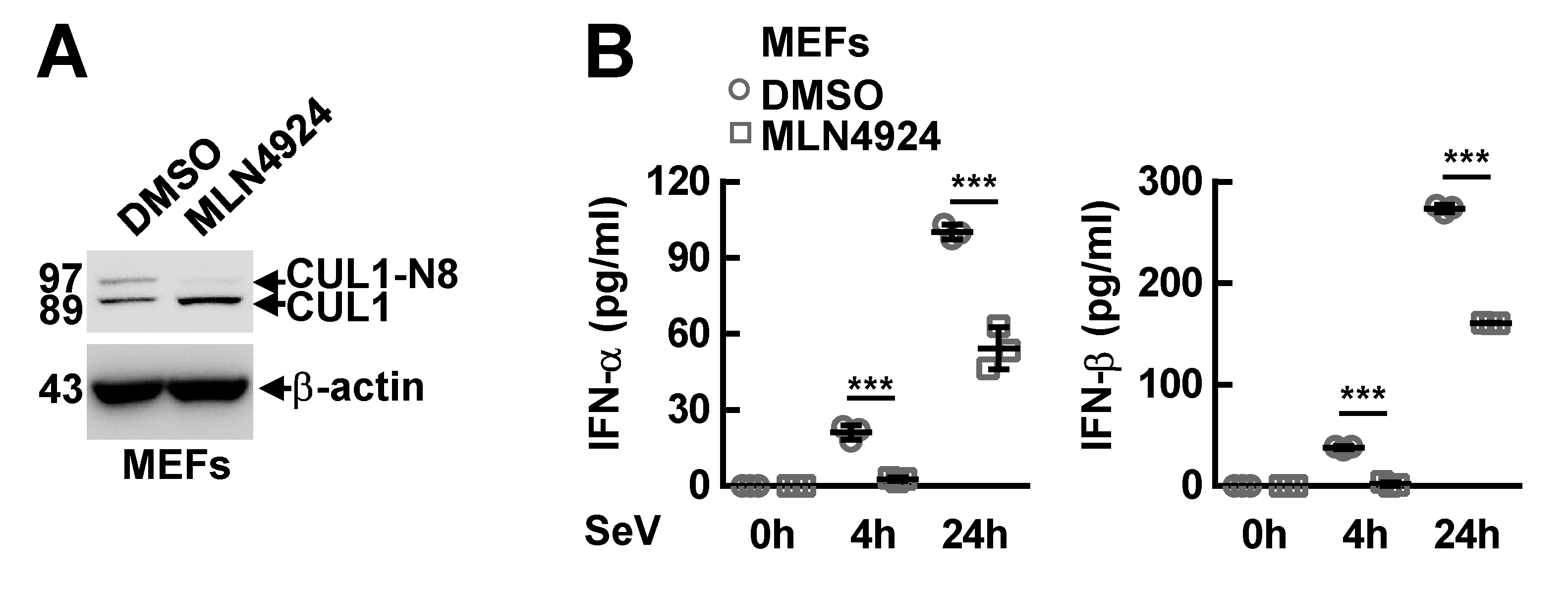

Supplement: S2 Fig — (A) IB analysis to confirm the efficiency of MLN4924 pretreatment (0.5 μM, 3 h) in MEFs. (B) After MLN4924 pretreatment (0.5 μM, 3 h), MEFs were infected with SeV for the indicated time periods. Then the supernatants were subjected to ELISA. Quantitative data are shown as Mean ± SD (n = 3 per group). ***p< 0.001. (TIF) [file ppat.1009901.s002.tif]

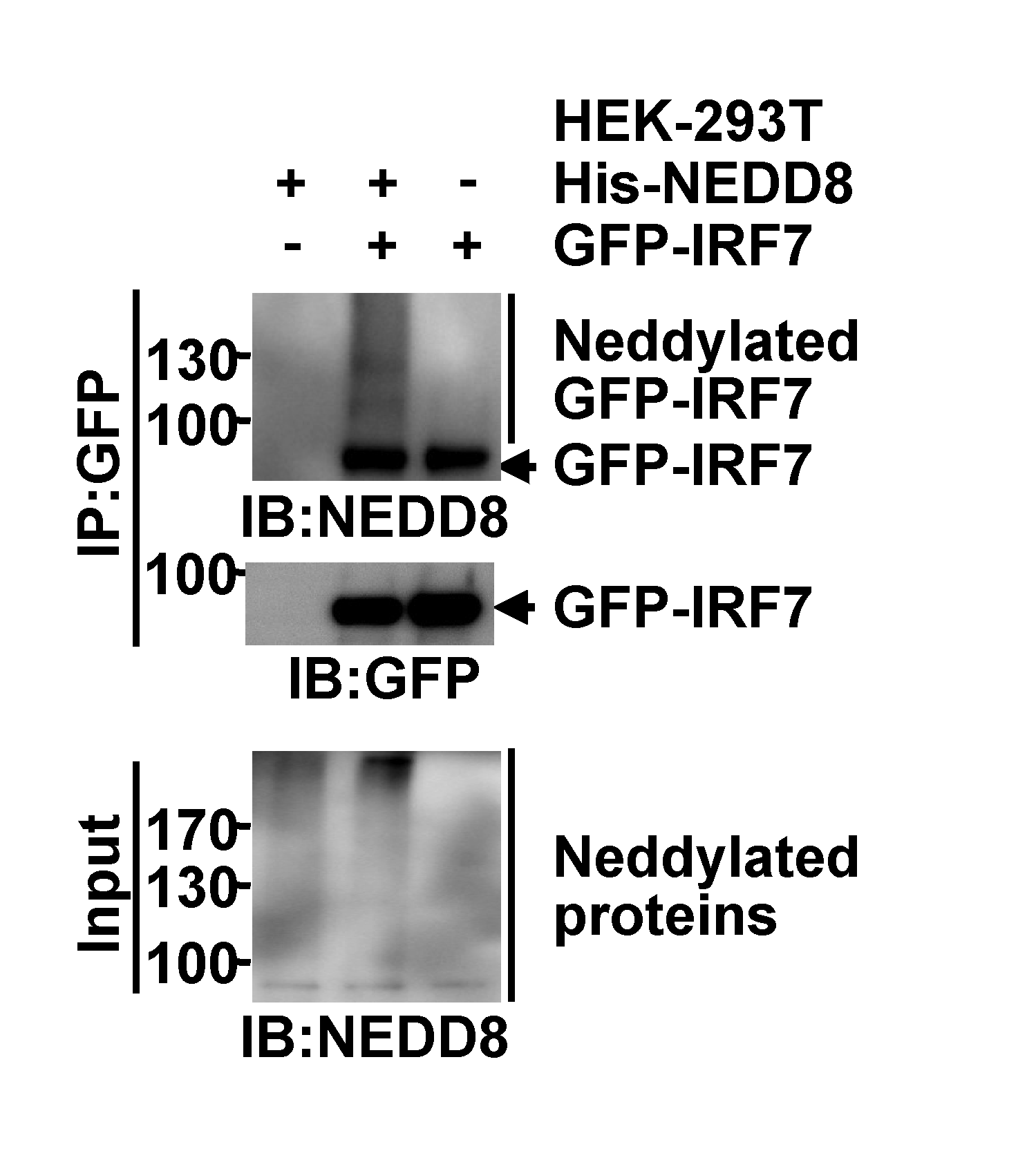

Supplement: S3 Fig — HEK-293T cells were transfected with the indicated mammalian expression vectors. Twenty-four hours later, possible neddylation of exogenous murine IRF7 was examined by IB analysis with the indicated antibodies after IP under partially denaturing conditions with an antibody against GFP. (TIF) [file ppat.1009901.s003.tif]

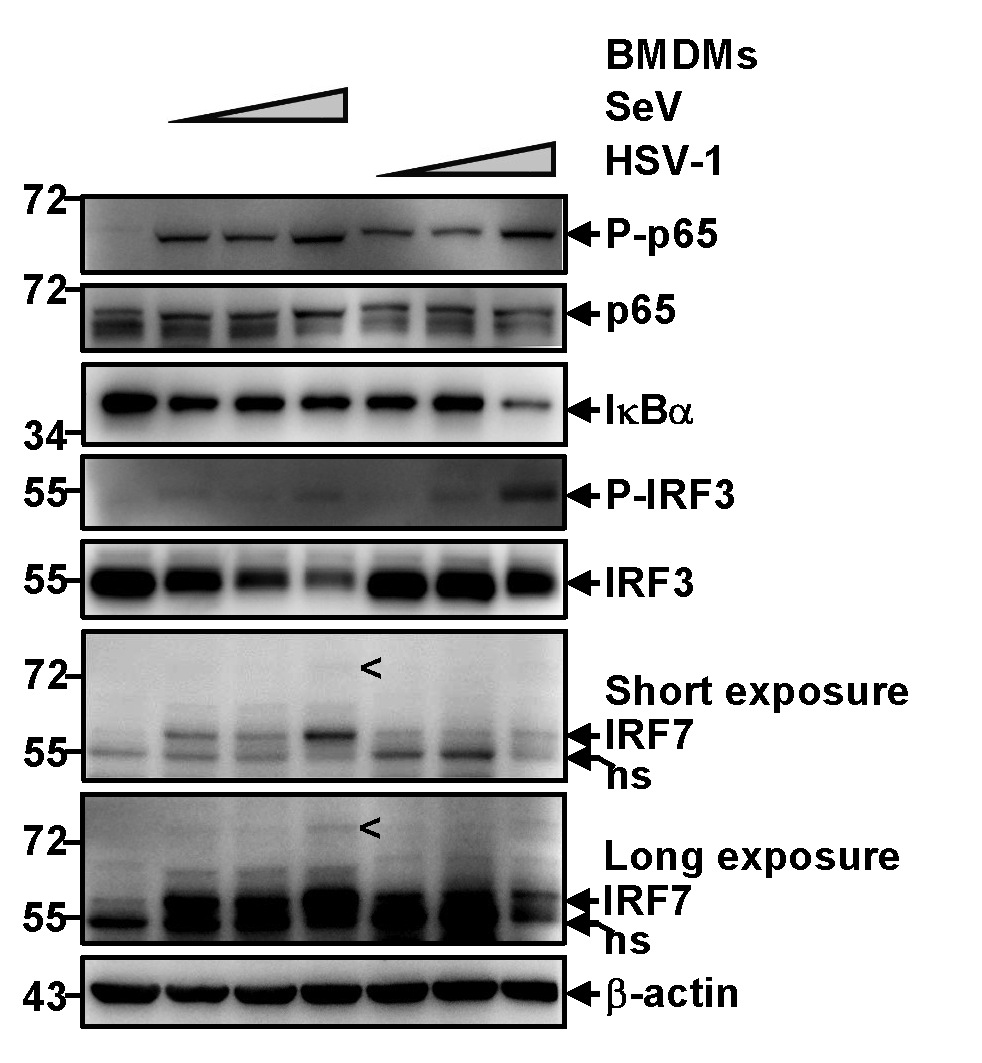

Supplement: S4 Fig — WT BMDMs were infected with different doses of the indicated viruses for 6 h. Cell lysates were then harvested and subjected to IB analysis with the indicated antibodies. P-p65, phosphorylated p65 at Ser536; P-IRF3, phosphorylated IRF3 at Ser396; ns, non-specific band. (TIF) [file ppat.1009901.s004.tif]

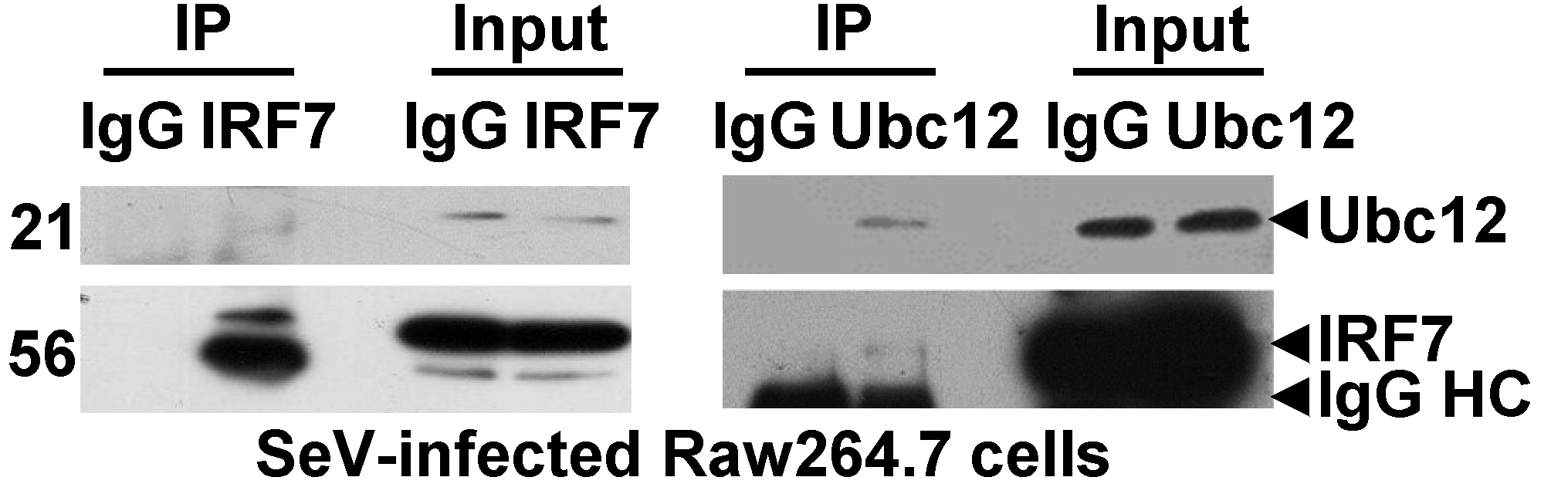

Supplement: S5 Fig — IB analysis of the interaction between endogenous IRF7 and endogenous Ubc12 in SeV-infected Raw264.7 after IP with an anti-IRF7 antibody (Left) or an anti-Ubc12 antibody (Right). Control antibody: rabbit IgG; IgG HC, IgG heavy chain; ns, non-specific. (TIF) [file ppat.1009901.s005.tif]

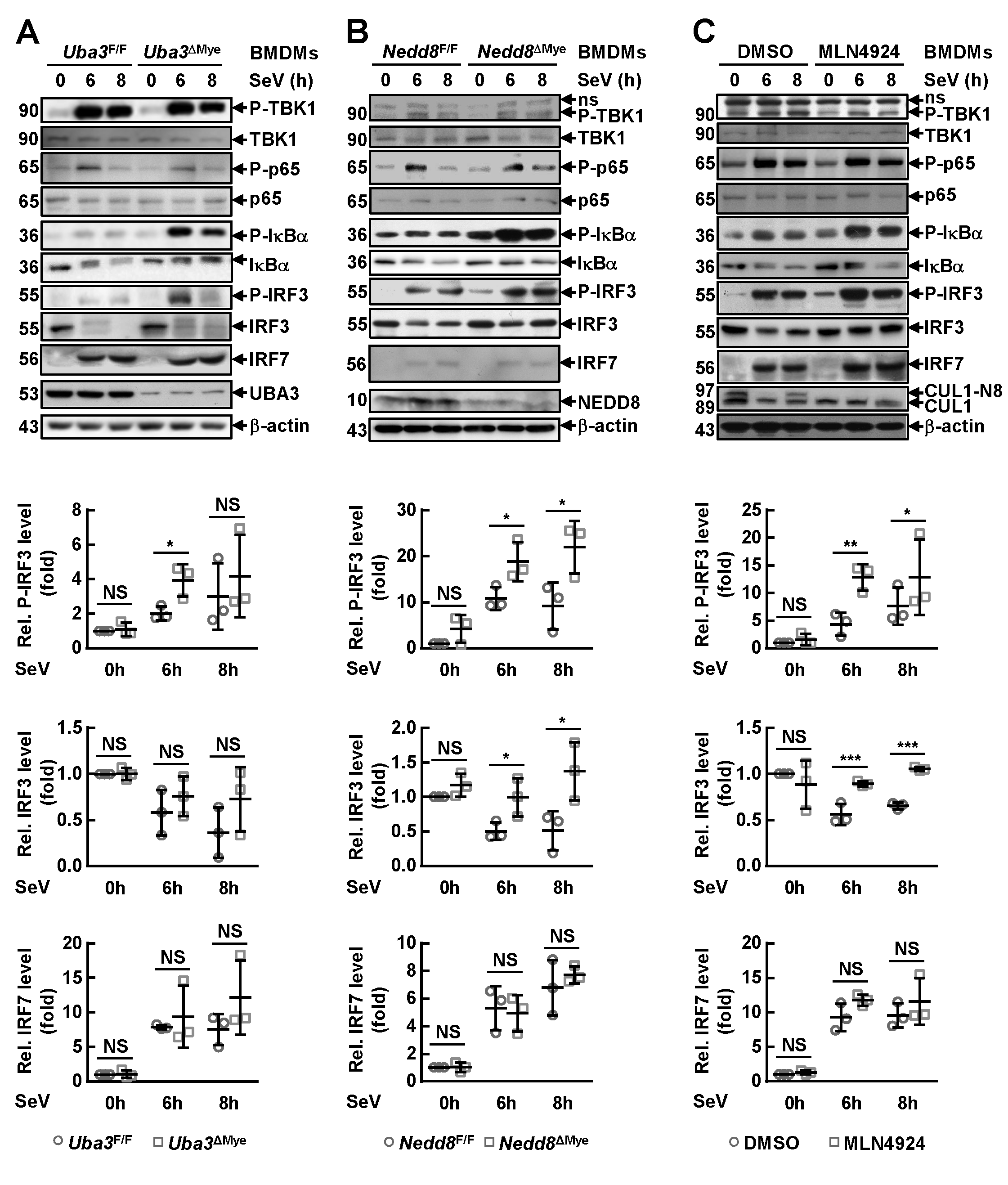

Supplement: S6 Fig — (A-C) After BMDMs were cultured from the indicated mouse models (A-B) or WT BMDMs were pretreated with 0.1 μM MLN4924 for 3 h (C), the cells were infected with SeV for the indicated time periods. Cell lysates were then harvested and subjected to IB analysis with the indicated antibodies (Top). P-TBK1, phosphorylated TBK1 at Ser172; P-p65, phosphorylated p65 at Ser536; P-IκBα, phosphorylated IκBα at Ser32; P-IRF3, phosphorylated IRF3 at Ser396; ns, non-specific band. The density of the indicated bands was quantified by scanning densitometry and normalized to β-actin (Bottom). Quantitative data are shown as Mean ± SD (n = 3 per group). *p< 0.05; **p< 0.01; ***p< 0.001; NS, not significant. (TIF) [file ppat.1009901.s006.tif]

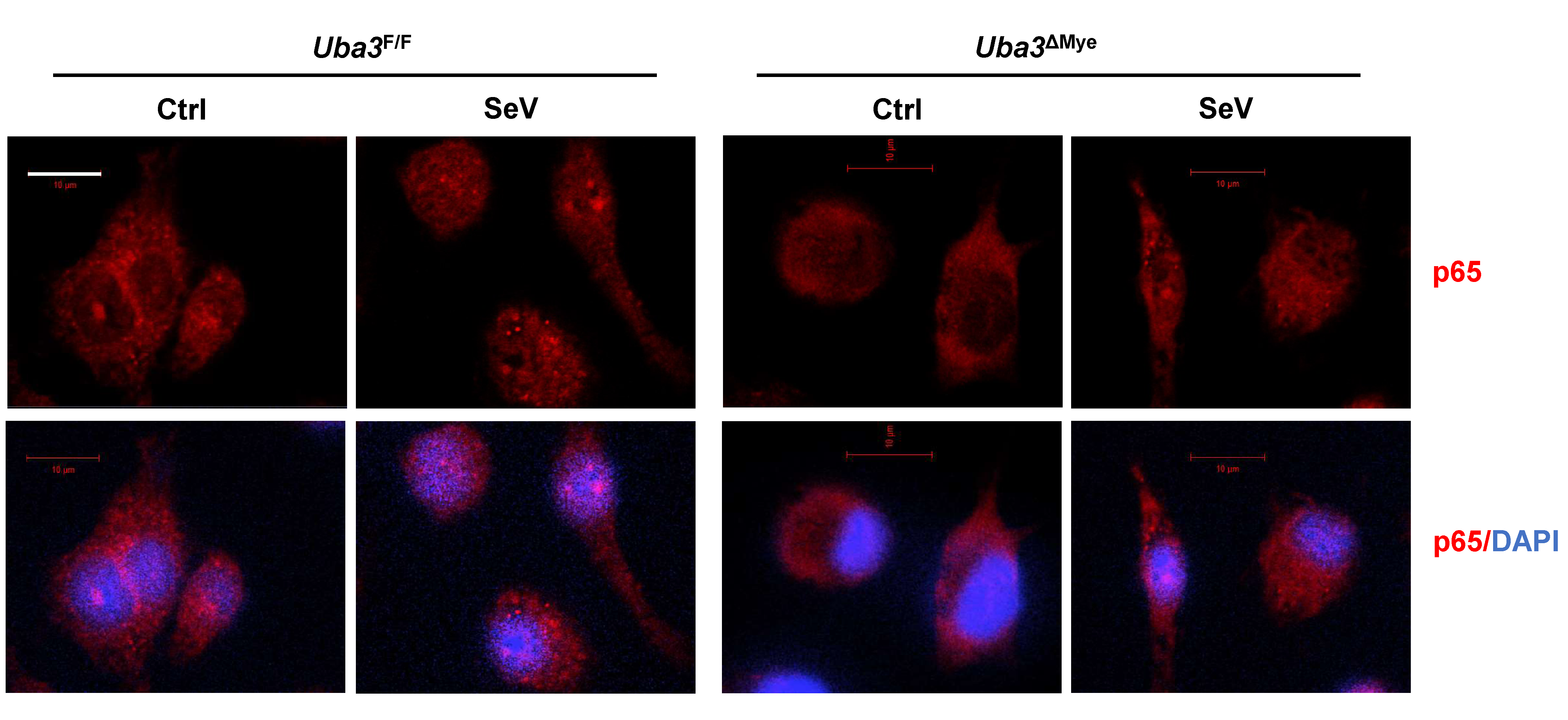

Supplement: S7 Fig — Six hours after BMDMs from Uba3F/F and Uba3ΔMye mice were infected with SeV or left uninfected, the nuclear translocation of NF-κB was examined by indirect immunofluorescence analysis with an antibody against p65 (Scale bar, 10 μm). (TIF) [file ppat.1009901.s007.tif]

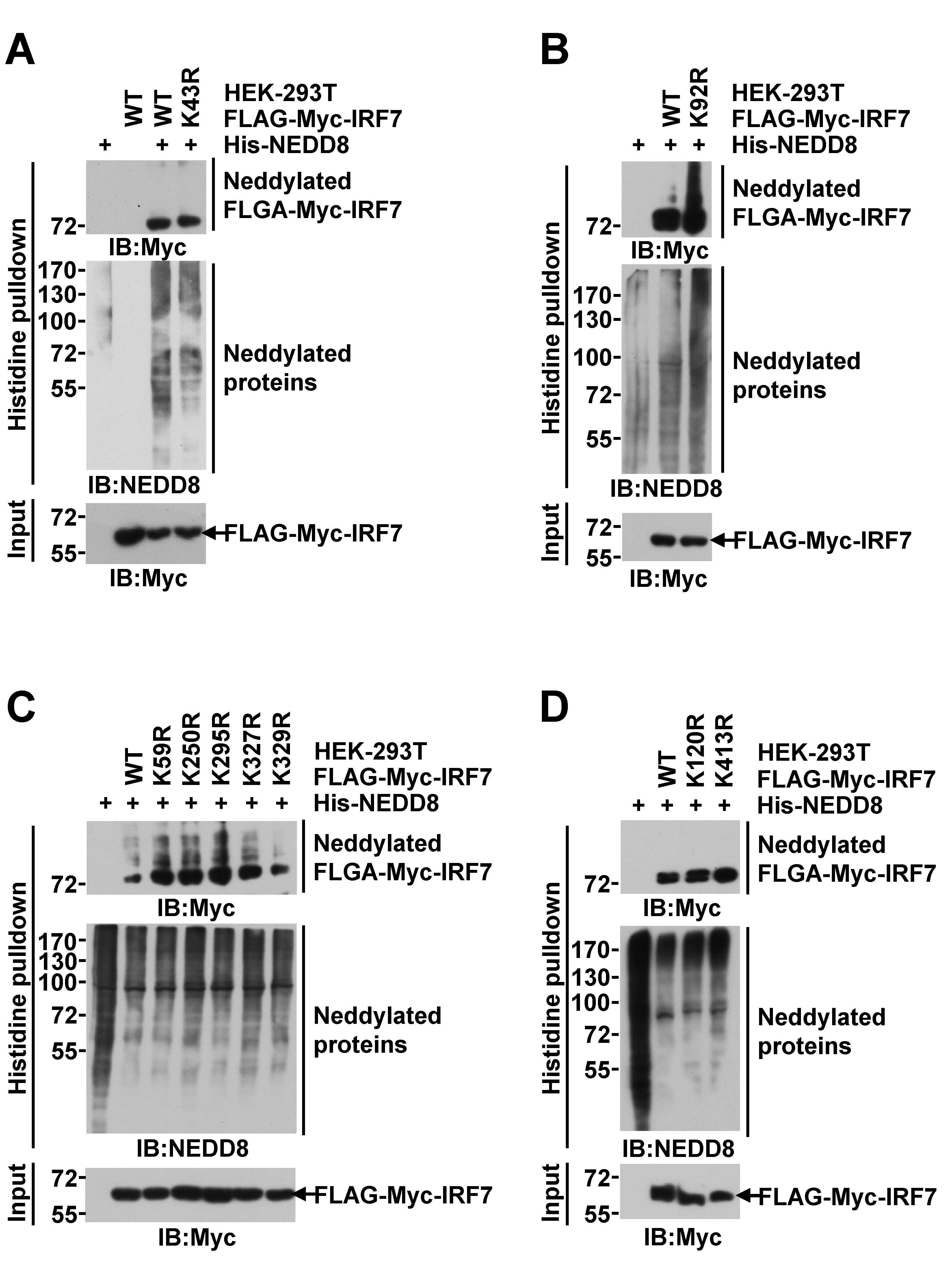

Supplement: S8 Fig — HEK-293T cells were transfected with mammalian expression vectors encoding His-NEDD8 and FLAG-Myc-tagged murine IRF7 WT or mutants. Twenty-four hours later, neddylation of exogenous murine IRF7 was examined by IB analysis with the indicated antibodies after histidine pulldown under fully denaturing conditions. (TIF) [file ppat.1009901.s008.tif]

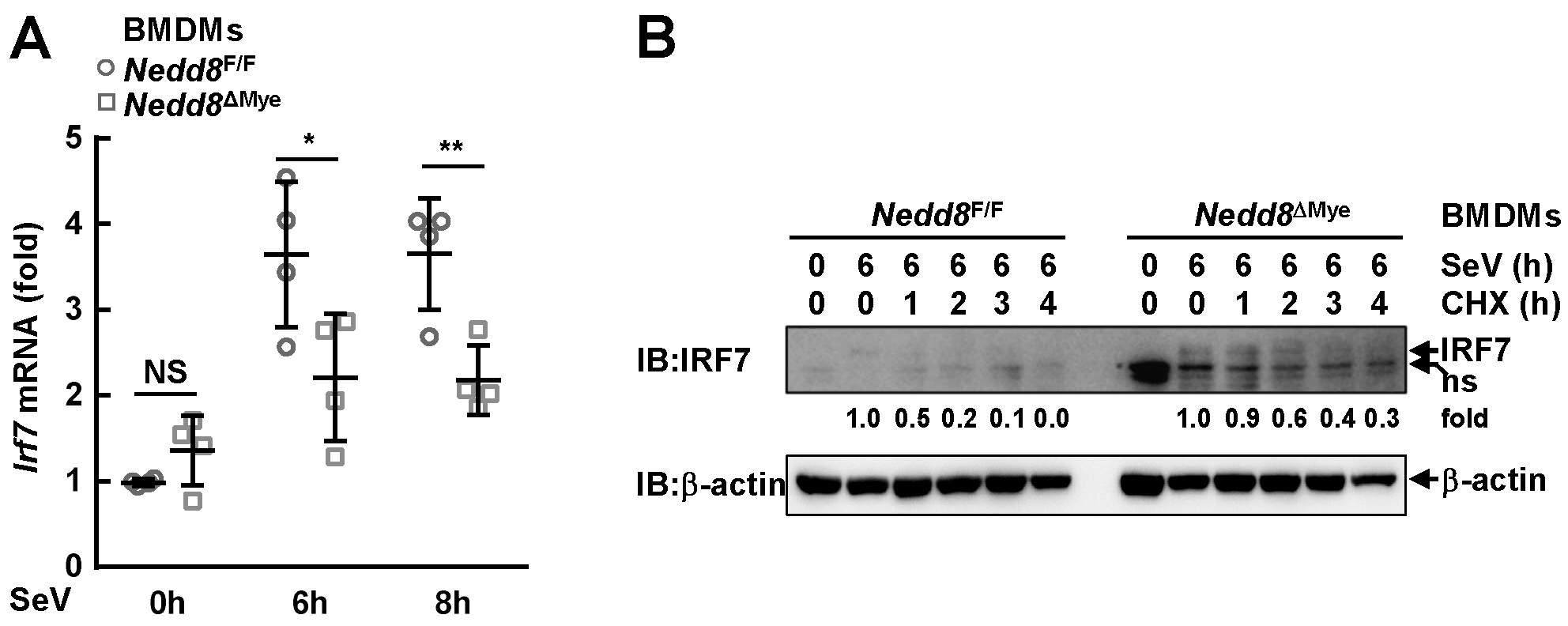

Supplement: S10 Fig — (A) BMDMs from Nedd8F/F and Nedd8ΔMye mice were infected with SeV for the indicated time periods. Then cells were subjected to quantitative RT-PCR analysis. Data are shown as Mean ± SD (n = 4 per group). *p< 0.05; **p< 0.01; NS, not significant. (B) After BMDMs from Nedd8F/F and Nedd8ΔMye mice were infected with or without SeV for 6 h, the cells were treated with 10 μg/mL cycloheximide (CHX) for various periods of time. Then, the half-life of IRF7 was analyzed by IB. ns, non-specific band. Densitometric readings are shown for IRF7 and normalized to β-actin. (TIF) [file ppat.1009901.s010.tif]

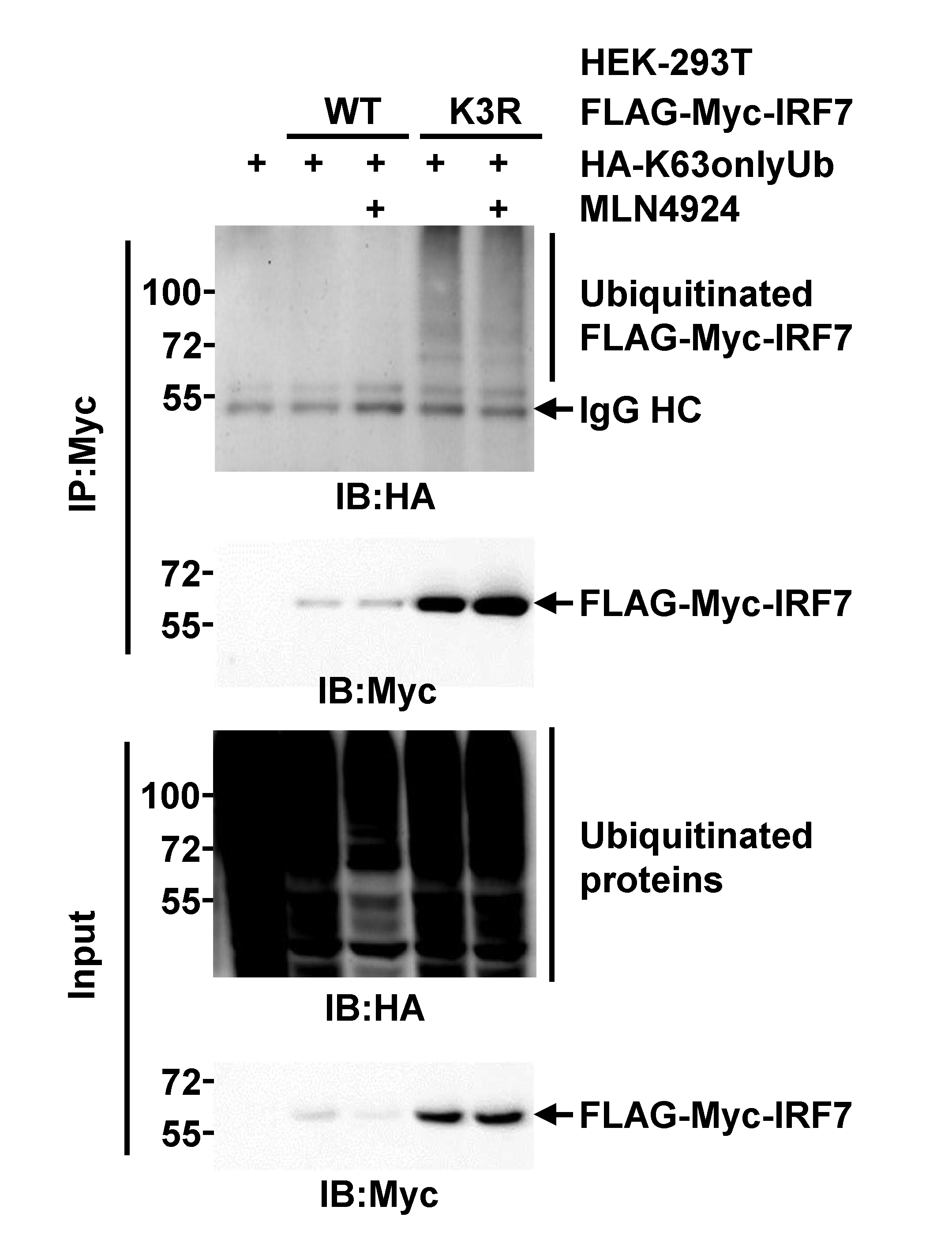

Supplement: S11 Fig — HEK-293T cells were transfected with mammalian expression vectors encoding HA-K63onlyUb and FLAG-Myc-tagged murine IRF7 WT or K3R mutant. After 12 h, cells were treated with 0.5μM MLN4924 for another 24 h or left untreated. The modification of exogenous murine IRF7 was then examined by IB analysis with the indicated antibodies after IP with an antibody against Myc-tag. IgG HC, IgG heavy chain. (TIF) [file ppat.1009901.s011.tif]

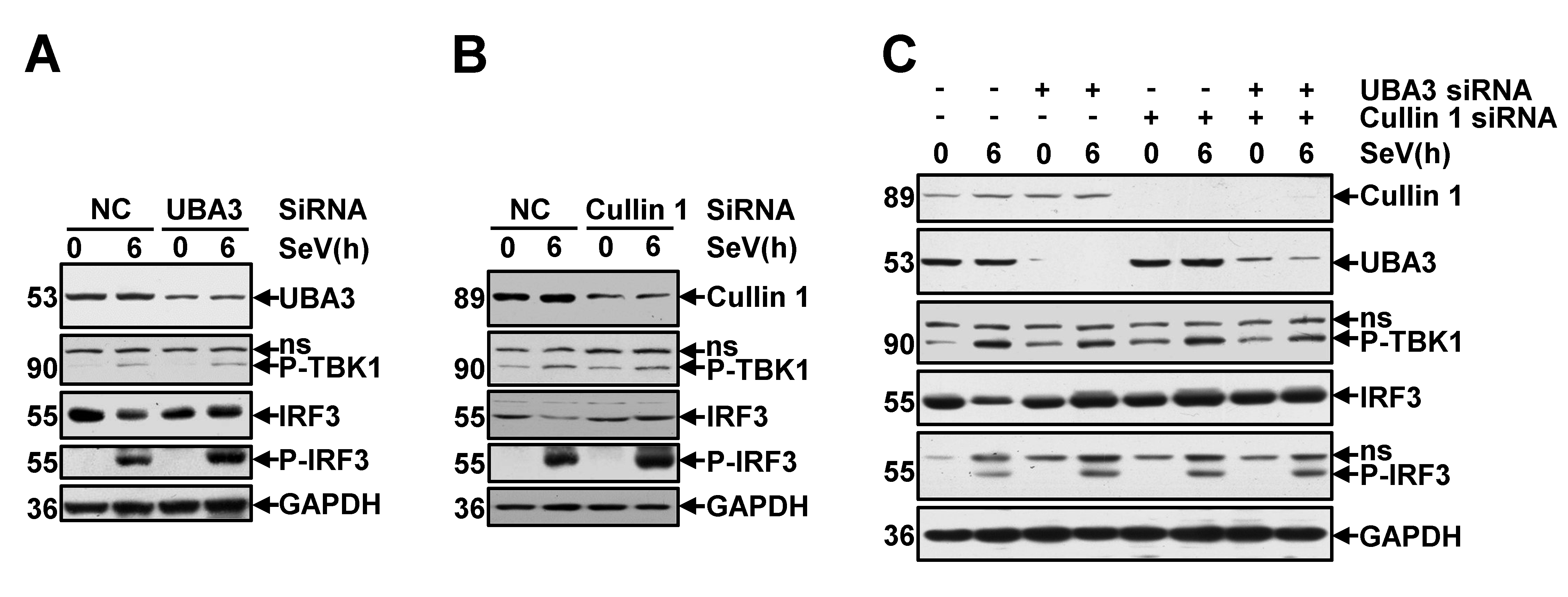

Supplement: S12 Fig — Forty-eight hours after MEFs were transfected with the indicated siRNAs, cells were infected with SeV for the indicated time periods. Cell lysates were then harvested and subjected to immunoblotting analysis with the indicated antibodies. P-TBK1, phosphorylated TBK1 at Ser172; P-IRF3, phosphorylated IRF3 at Ser396; ns, non-specific band. (TIF) [file ppat.1009901.s012.tif]
