# Supplementary material for: Myeloid neddylation targets IRF7 and promotes host innate immunity against RNA viruses
Source: PLoS Pathog. 2021 Sep 10;17(9):e1009901. doi: 10.1371/journal.ppat.1009901 (PMC8432861; doi:10.1371/journal.ppat.1009901)
Supplement: S9 Fig — (PDF) [file ppat.1009901.s009.pdf]

[R].QGSPHYTIYLGFGQDLSAGRPKEKTLILVK.[L]

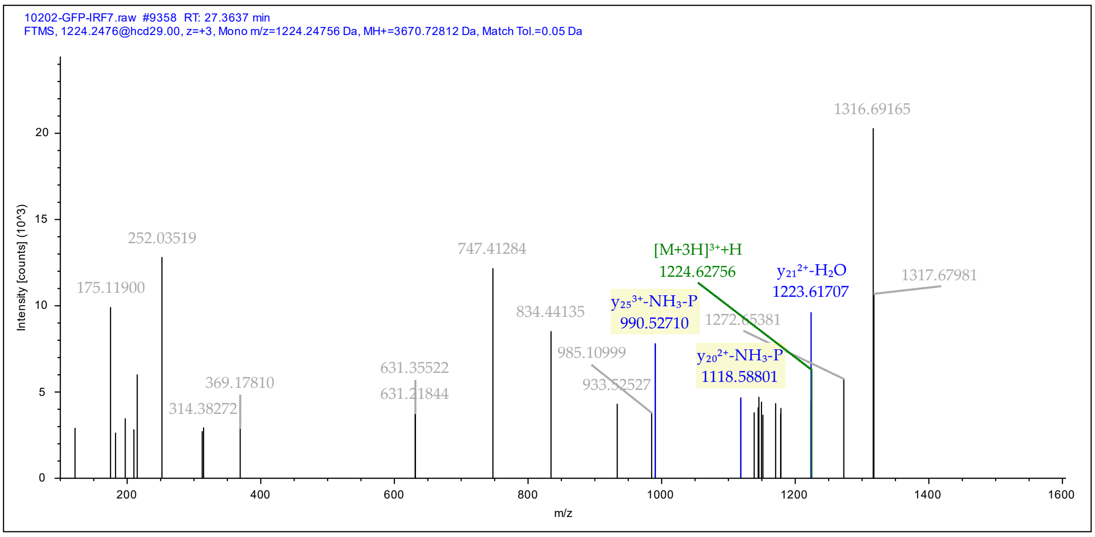

[R].QGSPHYTIYLGFGQDLSAGRPKEKTLILVK.[L]

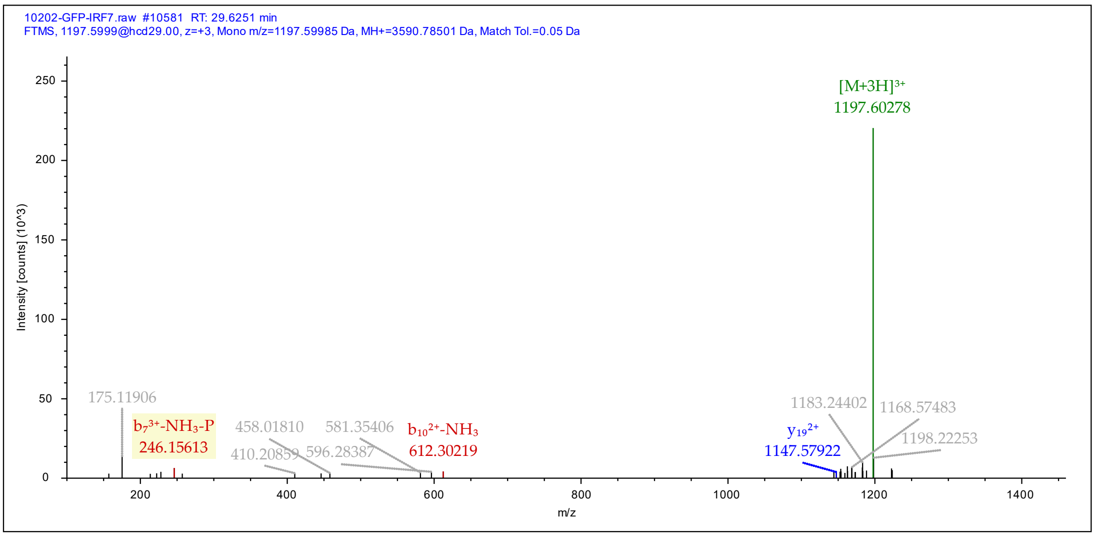

[R].RQGSPHYTIYLGFGQDLSAGRKEK.[T]

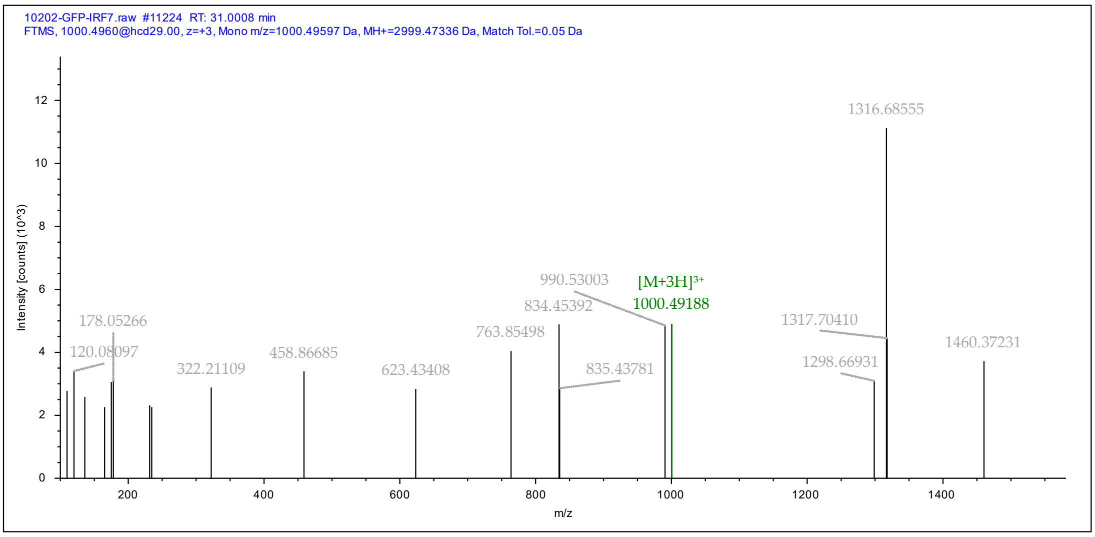

[R].MGKCKVYWEVGSPMGTTGPSTPPQLLER.[N]

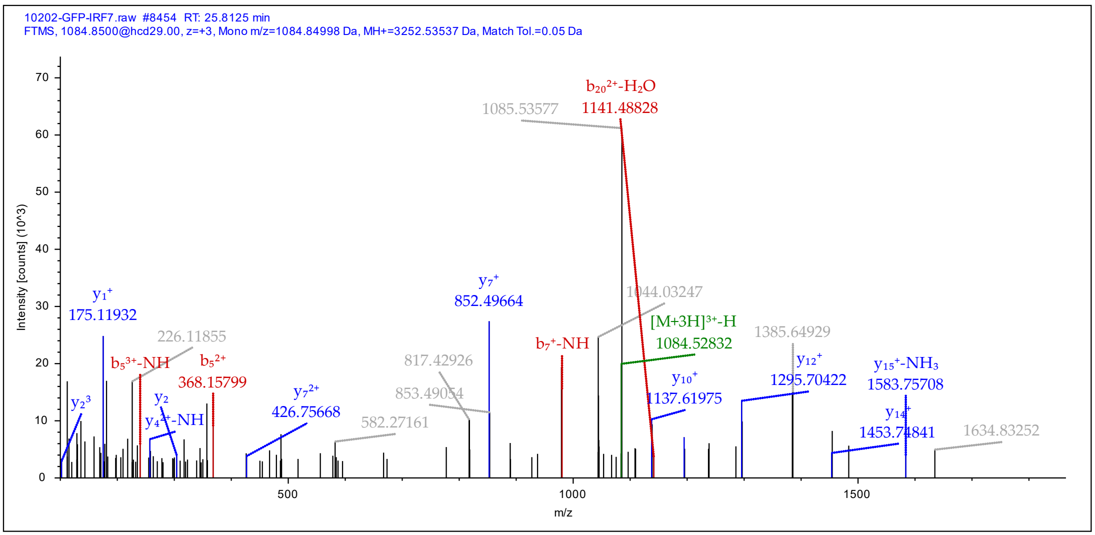

[K].CKVYYWEVGSPMGTTGPSTPPQLLER.[N]

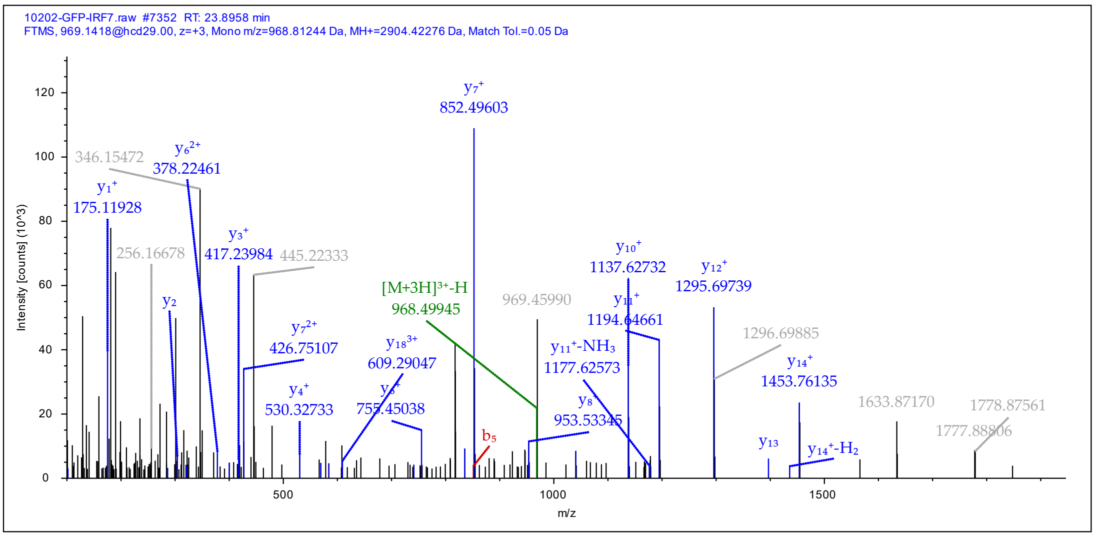

S9 Fig. Possible neddylated peptides of murine IRF7 as revealed by mass spectrometry analysis.
