# Supplementary material for: Myeloid neddylation targets IRF7 and promotes host innate immunity against RNA viruses
Source: PLoS Pathog. 2021 Sep 10;17(9):e1009901. doi: 10.1371/journal.ppat.1009901 (PMC8432861; doi:10.1371/journal.ppat.1009901)
Supplement: S1 Table — (PDF) [file ppat.1009901.s013.pdf]

S1 Table. Primers for quantitative RT-PCR.

Primer	Sequence (5’-3’)
Gapdh mRNA-forward	AGGTCGGTGTGAACGGATTTG
Gapdh mRNA-reverse	TGTAGACCATGTAGTTGAGGTCA
H1N1 RNA -forward	AAGACCAATCCTGTCACCTCTGA
H1N1 mRNA -reverse	CAAAGCGTCTACGCTGCAGTCC
Ifna1 mRNA-forward	GCCTTGACACTCCTGGTACAAATGAG
Ifna1 mRNA-reverse	CAGCACATTGGCAGAGGAAGACAG
Ifnb1 mRNA-forward	CCCTATGGAGATGACGGAGA
Ifnb1 mRNA-reverse	CCCAGTGCTGGAGAAATTGT
Irf7 mRNA-forward	CCCCAGCCGGTGATCTTTC
Irf7 mRNA-reverse	CACAGTGACGGTCCTCGAAG
Ifna1 promoter-forward	AAACACGGCTCTAAACTC
Ifna1 promoter-reverse	ATGCCTTAAATAGGGAAC
Ifna4 promoter-forward	AGATATGCCAAATCTGTG
Ifna4 promoter-reverse	GCTTTCTTTATGCACTCT
Ifna6 promoter-forward	GTCCTCCAAAGCTCAATT
Ifna6 promoter-reverse	CCTCCAGTAAGCCATCTA
