# Supplementary material for: Myeloid neddylation targets IRF7 and promotes host innate immunity against RNA viruses
Source: PLoS Pathog. 2021 Sep 10;17(9):e1009901. doi: 10.1371/journal.ppat.1009901 (PMC8432861; doi:10.1371/journal.ppat.1009901)
Supplement: S2 Table — (PDF) [file ppat.1009901.s014.pdf]

S2 Table. Primary antibodies used in this study.

Antibody	Host species	Supplier	Catalog No.	Application
β-actin	Mouse	Santa Cruz Biotechnology	sc-8432	IB
CBP	Rabbit	Abclonal (Wuhan, China)	A14237	IB
Cullin 1	Rabbit	Santa Cruz Biotechnology	sc-11384	IB
FLAG	Mouse	Sigma-Aldrich (St. Louis, MO, USA)	F1804	IB, IP
GAPDH	Mouse	Sungene Biotech (Shanghai, China)	KM9002	IB
GFP	Rabbit	MBL International (Woburn, MA, USA)	598	IB, IP
HA	Rabbit	Proteintech (Chicago, IL, USA)	51064-2-AP	IB
IRF3	Rabbit	Proteintech	11312-1-AP	IB
IRF7	Rabbit	Cell Signaling Technology	72073	IB
IRF7	Rabbit	Home-made	----	IP
IκBα	Rabbit	Santa Cruz Biotechnology	sc-371	IB
Myc	Mouse	MBL International	M192-3	IB, IP
NEDD8	Rabbit	Abcam (Cambridge, UK)	ab81264	IB
p65	Rabbit	Cell Signaling Technology	8242	IB
P-p65 (Ser536)	Rabbit	Cell Signaling Technology	3033	IB

P-IRF3 (Ser396)	Rabbit	Cell Signaling Technology	4947	IB
P-IRF7 (Ser437/438)	Rabbit	Cell Signaling Technology	24129	IB
P-IκBα (Ser32)	Rabbit	Cell Signaling Technology	2859	IB
P-TBK1 (Ser172)	Rabbit	Cell Signaling Technology	5483	IB
TBK1	Rabbit	Abclonal	A2573	IB
UBA3	Rabbit	Abcam	ab124728	IB
Ubc12	Rabbit	Proteintech	14520-1-AP	IB, IP
